# Pharmacological Efficacy of *Tamarix aphylla*: A Comprehensive Review

**DOI:** 10.3390/plants11010118

**Published:** 2021-12-31

**Authors:** Saad Ali Alshehri, Shadma Wahab, Shahabe Saquib Abullais, Gotam Das, Umme Hani, Wasim Ahmad, Mohd Amir, Ayaz Ahmad, Geetha Kandasamy, Rajalakshimi Vasudevan

**Affiliations:** 1 Department of Pharmacognosy, College of Pharmacy, King Khalid University, Abha 61421, Saudi Arabia; salshhri@kku.edu.sa; 2 Department of Periodontics and Community Dental Sciences, College of Dentistry, King Khalid University, Abha 61421, Saudi Arabia; drsaquib24@gmail.com; 3 Department of Prosthodontics, College of Dentistry, King Khalid University, Abha 61421, Saudi Arabia; gmenghwar@kku.edu.sa; 4 Department of Pharmaceutics, College of Pharmacy, King Khalid University, Abha 62529, Saudi Arabia; ummehaniahmed@gmail.com; 5 Department of Pharmacy, Mohammed Al-Mana College for Medical Sciences, Safaa, Dammam 34222, Saudi Arabia; wasimahmadansari@yahoo.com (W.A.); 1980ayaz@gmail.com (A.A.); 6 Department of Natural Products and Alternative Medicines, College of Clinical Pharmacy, Imam Abdulrahman Bin Faisal University, P.O. Box 1982, Dammam 31441, Saudi Arabia; matahmad@iau.edu.sa; 7 Department of Clinical Pharmacy, College of Pharmacy, King Khalid University, Abha 61421, Saudi Arabia; glakshmi@kku.edu.sa; 8 Department of Pharmacology, College of Pharmacy, King Khalid University, Abha 61421, Saudi Arabia; raja@kku.edu.sa

**Keywords:** *T. aphylla*, phytochemicals, biological activity, anti-inflammatory, antidiabetic, antibacterial, antifungal, anticholinesterase

## Abstract

*Tamarix aphylla* is a well-known species of the genus *Tamarix*. *T. aphylla* (Tamaricaceae) is a perennial tree in Asia, the Middle East, and Central Africa. It is used as a carminative diuretic in tuberculosis, leprosy, and hepatitis. Various pharmacological properties have been shown by *T. aphylla,* such as antidiabetic, anti-inflammatory, antibacterial, antifungal, anticholinesterase, and wound-healing activity. However, *T. aphylla* has not received much attention for its secondary metabolites and bioactive constituents. Research has shown that this plant has hidden potential that needs to be explored. This review aims to cover botanical classification, geographical distribution, taxonomy, ethnobotanical uses, and the phytochemical compounds found in *T. aphylla*. The toxicology and pharmacological effects of *T. aphylla* are also discussed. We examined various scholarly resources to gather information on *T. aphylla*, including Google Scholar, Scopus, Science Direct, Springer Link, PubMed, and Web of Science. The finding of this work validates a connection between *T. aphylla* in conventional medicine and its antidiabetic, antibacterial, anti-inflammatory, wound-healing, antifungal, anticholinesterase, and other biological effects. *T. aphylla’s* entire plant (such as bark, leaves, fruits) and root extracts have been used to treat hypertension, stomach discomfort, hair loss, cough and asthma, abscesses, wounds, rheumatism, jaundice, fever, tuberculosis, and gum and tooth infection. The phytochemical screening revealed that noticeably all extracts were devoid of alkaloids, followed by the presence of tannins. In addition, different parts have revealed the existence of steroids, flavonoids, cardiac glycosides, and byproducts of gallic acid and ellagic acid. *T. aphylla* has shown many valuable activities against different diseases and supports its traditional uses. Therefore, high-quality preclinical research and well-designated clinical trials are needed to establish the efficacy and safety of this plant in humans.

## 1. Introduction

Natural product-based medication has been a source of treatment for various ailments for thousands of years [1]. Herbal medicines have long played a vital role in developing drugs to treat a variety of diseases. Currently, available medicines are distinctions or reproductions of constituents, combinations, and dilutions that exist in nature [2]. People’s interest in herbal medicines is currently increasing due to fewer or no side-effects [3]. Despite the minimal risk of adverse effects, the possibility of a medication interaction cannot be rejected. Another appealing attribute for accepting a comprehensive collection of herbal medications is cost-effectiveness. The use of herbal medicines is historical, but there is a space in ancient traditional practices fulfilled by alternative medicine and complementary medicines incorporating new technologies [4,5,6]. Therefore, we should continue to research new plant-based therapy from the vast reservoir of nature [7,8].

**Figure 1:**
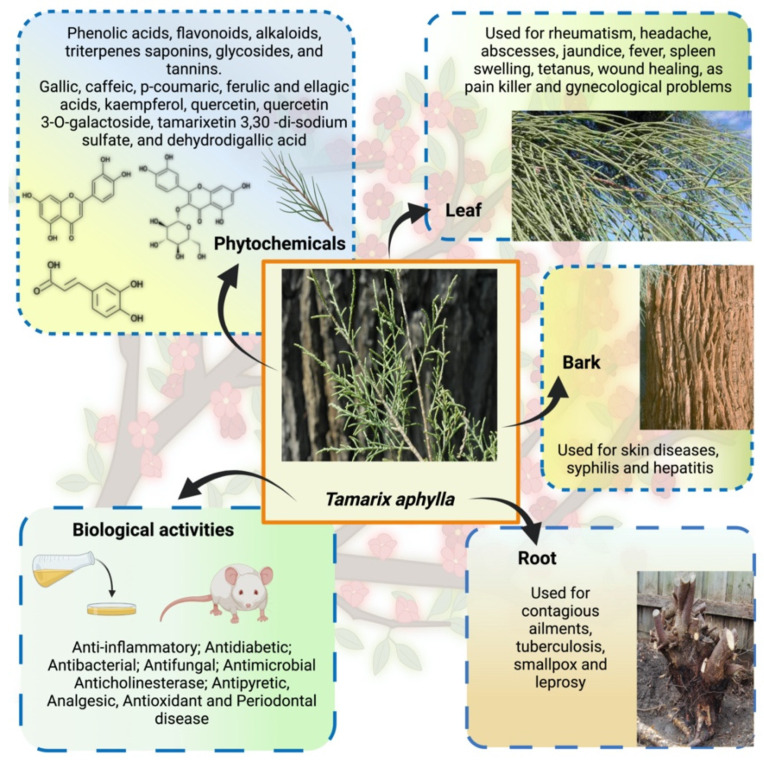
Overview of the *T. aphylla* ethnobotanical, phytochemical, and pharmacological potential.

Tamaricaceae (Caryophyllales) is a family of around 80 rheophytes, halophytes, and xerophytes grown in semi-arid and dry regions, especially in central and southwest Asia [9,10]. It is a native plant of African and Asian countries used by the locals for medicinal purposes and is commonly called tamarisk [11,12]. This plant has needle-like leaves coated in salt secreted by the salt glands [13]. The leaf attachment on the stem and the shape of the androecium are the most significant distinguishing features of *Tamarix* species: shrubs and trees. Some morphological characteristics have intermediate states of *Tamarix*; therefore, it is the most taxonomically challenging genus among angiosperms [9,10]. The reported phytochemical work on *Tamarix* species has shown that the main chemical constituents are polyphenolic constituents such as flavonoids, tannins, and phenolic acids [12]. The phytochemical research revealed that all extracts were noticeably devoid of alkaloids, followed by the presence of tannins. In addition, different parts of the plant have reported flavonoids, steroids, cardiac glycosides, and derivatives of ellagic acid and gallic acid [14,15,16,17,18,19]. *T. aphylla* has long been used as an antipyretic, analgesic, antirheumatic, and anti-inflammatory. Gall extract has been used for throat infections and temporarily constricting the vaginal mucous membrane before intercourse [4,14,20,21,22]. The bark of this plant is used to cure skin-related diseases, and gall on the flowers is used for its astringent effect [23]. There are various folk uses such as carminative, eczema, antimicrobial, antioxidant, aphrodisiac, anthelmintic, diuretic, anti-hemorrhoid, antidiarrheal, and skin diseases. In addition, many other studies have exhibited activities toward inflammation, joint pain, and internal tumors [20,24]. A complete overview of the *T. aphylla’s* ethnobotanical, phytochemical, and pharmacological potential is shown in Figure 1.

In this work, we reviewed the available research on *T. aphylla* and determined whether modern investigation into the various properties such as antidiabetic, antibacterial, anti-inflammatory, wound-healing, antifungal, and anticholinesterase activities of this plant validates its use to treat these diseases. This article reviews past *T. aphylla* research findings and identifies knowledge gaps that need to be addressed to fully grasp the mechanism of action of *T. aphylla* extracts in the treatment of various illnesses. In addition, the review covers phytochemistry and toxicological studies, highlighting the need for more research to back up the existing data and find new applications for this intriguing plant.

## 2. Material Methods

In the current review article, the information was collected from a literature search using various computerized databases such as PubMed, Google Scholar, Scopus, ScienceDirect, and Saudi Digital Library. Keywords such as *Tamarix aphylla*, *T. aphylla*, Tamarisk, *Tamarix* spp, Tamarix ethnomedicinal use, and ethnopharmacological aspects were used to search literature. In addition, keywords antimicrobial activity, biological activity, pharmacological properties, antifungal, cytotoxicity, anti-inflammatory, phytochemistry, phytochemical components, toxicology, anticholinesterase activity, and anticholinesterase effect with respect to *T. aphylla* were used to search literature. Furthermore, different phrases such as “genus *Tamarix* in chronic diseases”, “*Tamarix* species in chronic disease”, “*Tamarix aphylla* in diabetic disease”, “*Tamarix aphylla* in cytotoxicity”, “analgesic activity of *Tamarix aphylla*”, “anticholinesterase activity of *Tamarix aphylla*”, “*Tamarix aphylla* role in inflammatory diseases”, “*Tamarix aphylla* role in periodontal disease”, “antifungal activity of *Tamarix aphylla*”, “antibacterial activity of *Tamarix aphylla*”, and “wound-healing activity of *Tamarix aphylla*” were used to search the literature related to chronic diseases. In addition, further information was retrieved from various botanical books.

## 3. Ethnopharmacology: Traditional Practices

*T. aphylla* (Tamaricaceae) is the most well-known Tamarix species, reaching up to 18 m (60 feet) high. This plant is known with different names such as saltcedar, Athel tree, Athel tamarisk, and Athel pine. It is a treasure of various regions of the world including Central Africa, the Middle East, and Asia. Species of *Tamarix* grow in dry and hot climates, while some of its species are also found in moderate temperatures [12,13,25]. Ethnopharmacological reports on *T. aphylla* are presented in Table 1.

Most ethnopharmacological studies were published in Pakistan. These studies have shown that *T. aphylla* is used to treat various infectious diseases, including dental infections, smallpox, leprosy, tuberculosis, colds, and coughs [26,27,28]. Many other communications from other parts of the world have established that *T. aphylla* has antidiabetic, antibacterial, anti-inflammatory, and antifungal properties, in addition to being effective in periodontal disease, along with anticholinesterase and wound-healing activities. These activities are due to the tremendous number of phenolic compounds with astringent effects. *Tamarix aphylla,* together with the *Aerva javanica* plant, was studied ethnobotanically, chemically, and biologically in the Asir region of Saudi Arabia. The study claimed that both plants had a high usage value (UV), indicating knowledge of the medical relevance and uses of these plants. In the plains and valleys (wadis) of the Asir region, *T. aphylla* is ubiquitous, natural, and well-grown. *T. aphylla’s* different parts were reported to be used in various ailments, including joint pain, skin issues, and kidney problems. Plants with a high UV reflect their extensive use by locals in the area with several therapeutic effects toward human health issues. As a result, the author reported that bioactive phytochemicals and other pharmacological actions should be investigated in these plants [29].

Islamic religious scripture, Holy Quran, recognizes *T. aphylla* with names such as Athel or Tarfaa. As a useful traditional phytotherapy for jaundice, *T. aphylla* leaf ash is mixed with water, which is filtered and boiled after half an hour. Salt is left behind after water evaporation, of which 0.5–1 g is consumed with Shurbat-e-Bazoori twice daily [23]. Traditionally, the plant is known as “Mayyin Khurd” in Unani, Macheeka in Ayurveda, and Sivappattushavukku in Siddha, among other names. According to the history of folk medicinal usage, the plant has been used for antirheumatic, analgesic, and antipyretic purposes [4]. The folkloric assertion shows the use of *T. aphylla* in fever, inflammation, and pain, in addition to antimicrobial, antidiarrheal, anti-hemorrhoid, and antioxidant activities, along with use in eczema and skin diseases, as well as an aphrodisiac, diuretic, carminative, and anthelmintic [21,23,24].To cure wounds, abscesses, and rheumatism, the leaves are boiled in water, and water is strained; then, hot leaves are tied with the affected area. This therapy is continued for a week. The galls are astringent, and their aqueous extract is used as a gargle to cure throat infections. An extract of the leaves is used to treat toothache [14,30]. Ethnobotanical uses include the treatment of ulcer, GIT disorders, epilepsy, hair loss, and various dermatological problems [31,32]. There is a definitive history of using root decoctions to cure contagious ailments, such as smallpox, tuberculosis, and leprosy. The tree’s young branch and leaf decoction are used for tetanus, spleen, and gynecological problems [33].

*Tamarix aphylla* grows widely in diverse parts of the world; therefore, its vernacular names differ: German, Tamariske; Spanish, Taray; French, Tamaris; Arabic, Abal, Tarfaa, Ghaz, and Athel; Afrikaans, Woestyntamarisk; English, Athel pine, saltcedar, *Tamarix*, tamarisk, desert *Tamarix*, Athel tamarisk, and Athel tree [42,43,44].

## 4. Phytochemistry

This section of the review describes the structures of many secondary metabolites of *T. aphylla* and the functions of these metabolites in plants and humans. These compounds can be divided into four major biosynthetic classes: phenylpropanoids, terpenoids, alkaloids, and polyketides. The study of phytochemicals has led to drug discovery. The available literature on *T. aphylla* focuses on the characterization and isolation of particular parts of this plant [14,18,45,46]. A combination of phenolic chemicals characterizes this plant material [47]. There are several uses of *T. aphylla*, and its preparations are used as natural remedies to treat many diseases. Unfortunately, most of the studies have not thoroughly investigated the phytochemicals of *T. aphylla*. Most research has been conducted on the structural characterization and isolation of chemical components, including gallic acid, ellagic acid, and flavonoids [14,18,45,47,48,49]. Tannins and flavonoid-rich sources have been found in *T. aphylla* leaves [50].

Flavonoids and alkaloids extracted from *T. aphylla* have been examined for antibacterial activity. Phytochemical screening of most extracts of *T. aphylla* has exhibited the presence of tannins and a lack of alkaloids. Bioactive mixtures and metabolites have been found, such as cardiac glycosides, flavonoids, terpenoids, and steroids. Gallic and ellagic acid are derivatives of this plant species [15,16,17,19]. The leaves and stems of *T. aphylla* have a comparable phytochemical composition. Nevertheless, quantitative analyses have indicated that the leaves have a considerably greater quantity of polyphenols than the stems [17]. A floral extract of *T. aphylla* yielded isoferulic acid, 3-*O*-beta-glucopyranoside, glycosylated isoferulic acid, and novel phenolics such as dehydrodigallic acid dimethyl ester and tamarixetin 3,3’-disodium sulfate. According to the spectral data, their structures were developed [18]. Various studies have shown structural derivatives among hydrolyzable tannins [51]. *T. aphylla* galls, on the other hand, generate the ellagitannin tamarixellagic acid [49]. According to a study, under an abiotic stress environment, a halophytic plant stimulates oxidative stress reactive oxygen species in hostile conditions. Therefore, time and collection area are factors that may significantly change the amounts of secondary metabolites [17,49,52]. Table 2 lists a few important secondary metabolites of *T. aphylla*.

## 5. Pharmacological Efficacy of *Tamarix aphylla*

### 5.1. Anti-Inflammatory Activity

Inflammation is the body’s reaction to fight burns, injuries, toxic stimuli, and infections that emerge as anorexia, fever, and edema. Inflammation is the body’s first defense reaction that removes undesirable effects and commences the healing operations. This protective system is crucial for a healthy and normal physiological state. Inflammation can be acute or chronic [58,59]. Removing the damaged stimuli is the treatment of acute inflammatory conditions; however, unchecked acute inflammation can lead to chronic inflammation. A chronic inflammatory response can cause serious health problems, such as neurological and cardiovascular diseases and cancer [60]. Alzheimer’s disease, cardiovascular disease, metabolic disorders, allergies, malignancies, and autoimmune illnesses all have chronic inflammation as a contributing factor [61]. Anti-inflammatory medications, both nonsteroidal and steroidal, are the most prescribed treatments. In most documented cases, the long-term use of these medicines is hampered by their side-effects, necessitating the therapeutic use of less toxic solutions with fewer unpleasant responses [62,63]. Consequently, there is a requirement for effective, safe, and cost-effective alternative therapies.

**Figure 2:**
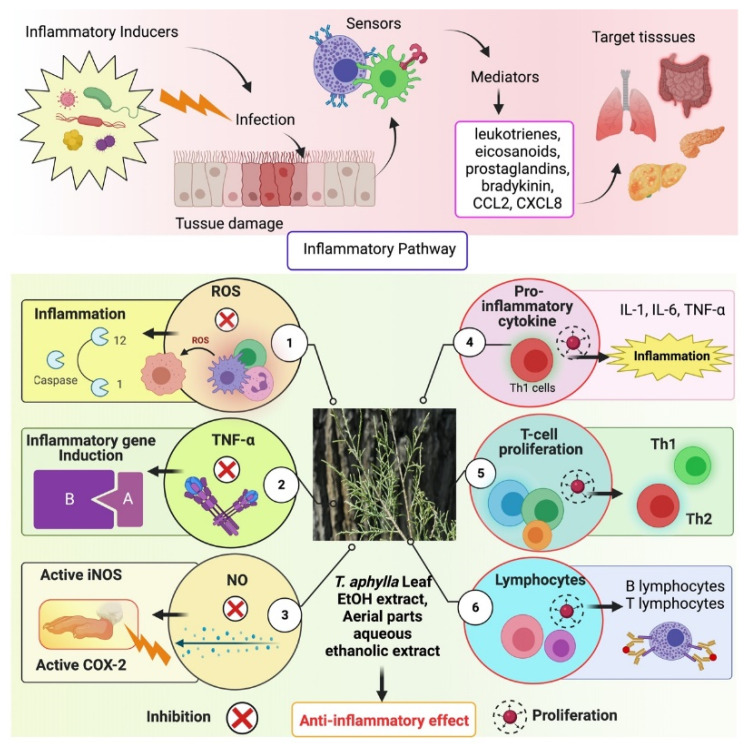
Inflammatory pathway and the probable role of *T. aphylla* as an anti-inflammatory agent. ROS: Reactive oxygen species, TNF-α: Tumor necrosis factor alpha, NO: Nitric oxide, IL-1: Interleukin-1, IL-6: Interleukin-6.

Herbal therapy has been used to treat various ailments since ancient times, including chronic inflammatory diseases. Medicinal plants contain anti-inflammatory properties and have limited or no adverse effects [3]. *T. aphylla* is generally safe and harmless to humans. However, inflammation is a significant development in many illnesses [64]. Consequently, there is a persistent need to learn more about the benefits of natural medications in treating inflammatory diseases to expand our scientific awareness. *Tamarix* species possess various compounds such as phenolic acids, tannins, sulfur-containing compounds, and flavonoids, which have been used since ancient times to cure chronic inflammatory disorders [11,12]. *T. aphylla* is an encouraging herbal medication with a wide range of metabolites, many medical uses, and pharmacological assurance. The aqueous ethanolic extract of *T. aphylla* galls has been shown to have anti-inflammatory and antipyretic properties [4]. The inflammatory pathway and probable role of *T. aphylla* as an anti-inflammatory drug are illustrated in Figure 2.

An investigation was carried out to examine the modulatory effect of *T. aphylla*, in which bioassay-guided isolation was carried out on several inflammatory indicators such as TNF-α and ROS, proliferation of lymphocyte T cells, and proinflammatory cytokines. The aqueous extract of *T. aphylla* suppressed intracellular ROS production, NO generation, and T-cell proliferation. The most effective inflammatory markers were the liquid–liquid fractional method partitioned aqueous ethanolic crude extract and *n*-BuOH and DCM extracts. In addition, 3,5-dihydroxy-4-methoxy benzoic acid methyl ester from the DCM extracts suppressed the generation of ROS and TNF-α. Furthermore, all mediators examined demonstrated inhibitory action in the presence of kaempferol. Therefore, *T. aphylla* constituents might be used as anti-inflammatory medications [63].

### 5.2. Wound-Healing Activity

The sequence of interconnected processes that replace damaged tissue at the injury site with newly produced tissue is called wound healing. Regular biological action is attained through four remarkably programmed stages: hemostasis, inflammation, proliferation, and remodeling. All four stages must happen in a certain time frame with a proper sequence to heal the wound. Many factors affect the process of wound healing, such as infection, oxygenation, sex hormones, age, stress, obesity, alcoholism, nutrition, smoking, and diabetes [65]. Various studies have proven that natural products affect all these factors. Natural therapeutics promote wound healing and fix impaired wounds with lessened side effects. Natural products have been used since ancient times to heal wounds due to their pharmacological importance, such as antibacterial, anti-inflammatory, and cell-stimulating potential [66]. The effectiveness of these natural products has been examined through in vitro, in vivo, and human studies. Skin wound healing is a perplexing biological activity and a coordinated cellular and biochemical sequence. Combining herbal materials with advanced drugs has improved regeneration [67].

**Figure 3:**
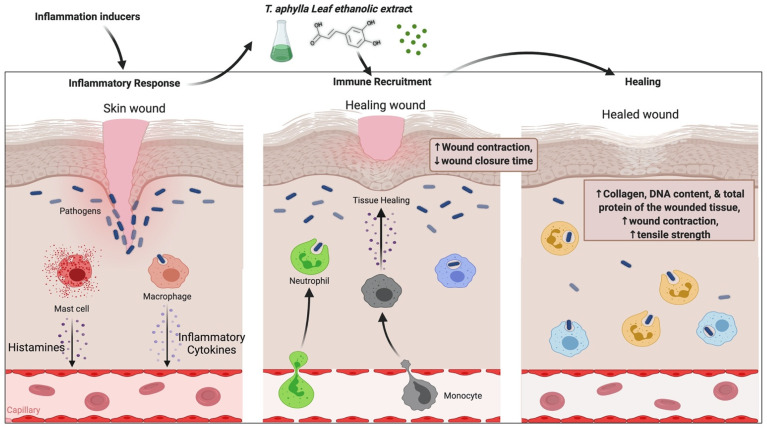
Probable mechanism of *T. aphylla* wound-healing activity.

*T. aphylla*’s anti-inflammatory and wound-healing properties are reported in Islamic literature and other resources from far-flung regions of Saudi Arabia. In the Al-Qassim region of Saudi Arabia, dried powders of all plant parts are used for 1 week to treat camel skin disorder (allergic or mycotic dermatitis) [68]. Burnt smoke from the dried pulverized plant leaves has history as an injury-healing agent and a dental painkiller [30]. It was found that boiling plant leaves and tying them to afflicted skin manages abscesses and rheumatism, in addition to wound healing [23]. An animal study examined an ethanolic extract of *T. aphylla* for wound-healing activity. A linear incision and a circular excision were employed to assess wound healing. Total protein estimation, antioxidant activity, lipid peroxides, uronic acid, hexosamine, and the tensile strength of tissue from incision wounds were examined. The period of epithelialization and TNF-α concentration of the wound tissues were also assessed in this study. Compared to conventional medication, the extract treatment groups exhibited considerable antibacterial activity. Results of the study showed that *T. aphylla* extract substantially enhanced the collagen content and remarkably increased tensile strength. *T. aphylla* extract has been shown to prevent oxidative damage, speed up healing activity, and have potent antioxidant properties. The epithelialization duration of the treated wounds was reduced by 40%. The study’s findings showed that the ethanolic extract of *T. aphylla* has strong wound-healing ability [69]. Another study investigated the wound-healing potential of *T. aphylla* extract by adopting the induced paw edema model in Wistar rats. In addition, the gel base of a *T. aphylla* extract, namely, Carbapol-934, was employed to investigate carrageen-induced anti-inflammatory properties. This examination showed that *T. aphylla* has wound-healing, anti-inflammatory, and antioxidant properties [70]. There is an urgent need to determine the actual mechanism of action, the safety, and the adverse effects of *T. aphylla* and its secondary metabolites; furthermore, there is a demand for revamped purification techniques, innovative extraction methods, and quality control evaluation with treatment regimes. Although *T*. *aphylla* is less expensive than current therapies, it is susceptible to geographic location, season, and batch-to-batch fluctuation, resulting in unanticipated allergic responses and adverse effects. The possible mechanism of *T. aphylla* wound-healing activity is shown in Figure 3.

### 5.3. Antibacterial Activity

In tropical and subtropical areas, human diseases, particularly those involving microorganisms such as bacteria, fungus, viruses, and nematodes, inflict significant harm. Over the last two decades, there has been a surge in interest in natural products as potential sources of novel antimicrobial agents. Consequently, various extracts from conventional herbal drugs have been examined to discover numerous pharmacological benefits [71,72,73]. Moreover, there is a need for novel herbal extracts and natural medicines that might effectively resist bacteria [71,74]. The antibacterial properties of plant phytochemicals have demonstrated their therapeutic usefulness in treating bacterial infections [75,76]. Therefore, documentation of the constituents and antibacterial activity of medicinal plants is precious since it may be utilized to conduct additional research to develop innovative antibacterial treatments.

Therefore, it is necessary to explore the phytochemical constituents of *T. aphylla* to treat infections [77]. This section aims to explore the antibacterial activities of extracts and secondary metabolites of *T. aphylla* to bridge existing research gaps, to provide suggestions based on the current understanding of *T. aphylla*’s advantages, and to advise more scientific research for innovative antibacterial treatments. All the secondary metabolites of *T. aphylla* listed in this review have medicinal properties, thus authenticating their usage [78]. For future study and the formation of new antibiotics to fight resistant pathogenic bacteria, all related studies of *T. aphylla* are discussed.

The antibacterial activity of a methanolic extract of *T. aphylla* bark against microbial strains in a study provided scientific evidence for the use of its phytoconstituents as an antimicrobial treatment. Halophytic species with stimulant and hepatotoxic properties are used to treat liver-related ailments. Halophytic species suppressed all bacterial strains, leaving *Listeria monocytogenes*, and studies have shown that polar methanolic fractions are more effective than polar chloroform fractions [79]. A study was conducted to test the antibacterial activity against 10 pathogenic bacteria implicated in severe ailments affecting humans and animals. Fresh and disease-free *T. aphylla* leaves were accumulated from Saudi Arabia’s various geographical locations. The antibacterial screening effectiveness of *T. aphylla* leaves against multidrug-resistant human infections was studied in vitro. The antibacterial effects of the methanol and ethanol leaf extracts were different and showed varying inhibitory effects against the examined pathogenic strains, ranging from modest inhibition (4 ± 0.6 mm) to high inhibition (20.7 ± 1.3 mm). There were no significant variations in the lowest inhibitory concentrations of ethanol and methanol extracts. *T. aphylla* contains antibacterial biomolecules that might be used to combat multidrug-resistant human infections [80]. The methanolic extract of *T. aphylla* and its phytochemical constituents were explored in vitro for antibacterial activity against various microbial strains. The disc diffusion assay with 25, 50, 75, and 100 mg/mL stock solutions was employed to examine the antibacterial activity toward *Escherichia coli*, *Bacillus subtilis*, *Salmonella typhi*, and *Staphylococcus aureus*, in addition to antifungal activity toward strains such as *Aspergillus flavus* and *Candida albicans*. The extract’s inhibitory efficacy in millimeters was calculated by measuring the zone of growth inhibition surrounding the discs and comparing it to the reference medication. The study showed that *T. aphylla* bark extract has a more significant inhibition zone at higher concentrations [81]. These compelling results can motivate health workers to use bioactive materials and phytoconstituents.

**Figure 4:**
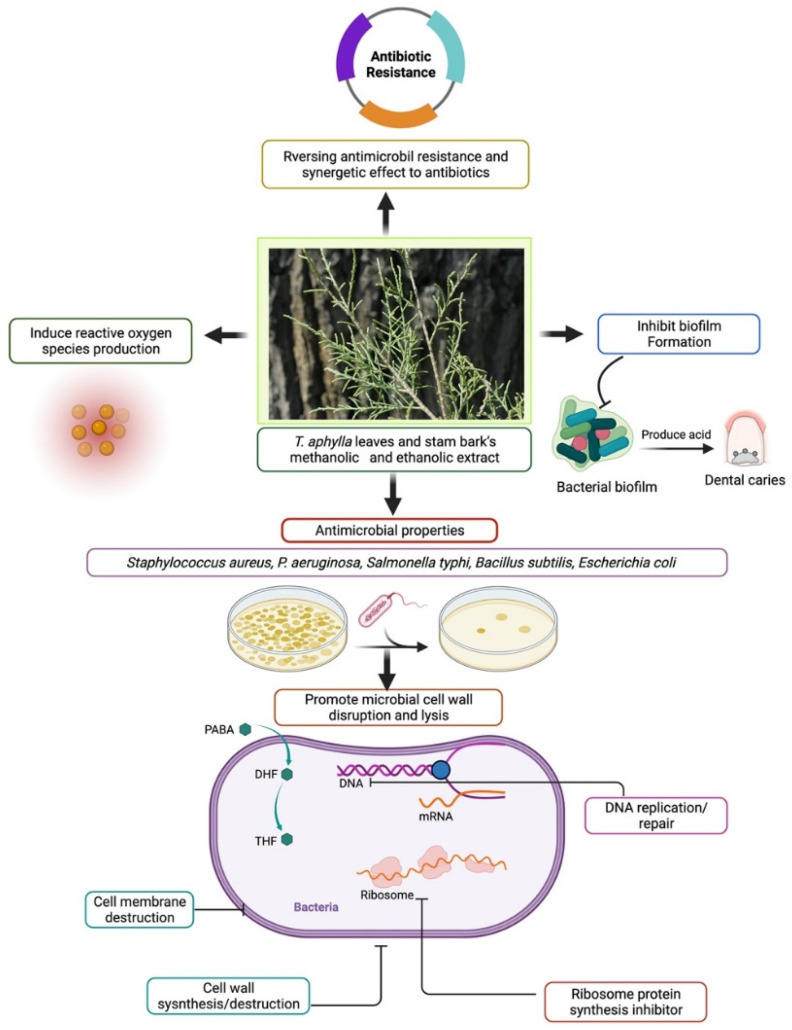
Possible antibacterial mechanism of *T. aphylla* against clinically important pathogens.

Biofilms are multispecies bacterial colonies that invade the oral cavity as plaque, causing dental cavities and periodontal disease [82]. In one study, three herbs were examined to determine their effectiveness against pinpointed dental biofilm-forming bacteria. Pathogenic strains that form dental biofilms were isolated, grown, and evaluated using PCR-based 16S ribosomal RNA (or 16S rRNA) nucleotide sequences. *T. aphylla* L., *Melia azadirachta* L., and *Acacia arabica* were the three crucial medicinal plants investigated to determine their antibacterial activity toward biofilm-forming strains. The findings showed that extracts of these three medicinal plants can be utilized against pathogenic bacteria that cause dental biofilms, and pharmaceutical companies should include them in dental care products [27]. The leaves of *T. aphylla* have been characterized as a rich source of tannins and flavonoids [50]. Kaempferol is a phytochemical found in *T. aphylla*. It has several therapeutic properties such as anticancer and antioxidant activities, while its usage is limited because of low permeability and poor solubility [83]. Kaempferol and resveratrol are two phenolic compounds that have shown antimicrobial activity. Therefore, researchers have employed two methods, time to kill and checkerboard, to evaluate this property. Studies showed that both compounds inhibited growth in MHB (Muller–Hilton broth) and milk according to the time-to-kill method [84]. In another study, *T. aphylla* suppressed the increase in microorganisms such as *S. aureus* and *P. aeruginosa* [69]. The possible antibacterial mechanism of *T. aphylla* is shown in Figure 4.

### 5.4. Antifungal Activity

Fungal infections are among the most dangerous diseases, killing around 1.5 million humans each year worldwide [85]. Infection of the skin ranks fourth among all fungal illnesses, and it also accounts for the bulk of deaths [86,87]. In the last several years, there has been an increase in the frequency of fungal illnesses and the resistance of specific fungus species to various fungicides used in medicine. Furthermore, fungi are among the most underappreciated diseases, while the reality is that amphotericin B and alternative commercially available antifungal therapies are still considered the gold standard. However, antifungal medicines have various side-effects such as toxicity, effectiveness, price, and extensive usage, resulting in resistant strains [88]. Resistance to antifungal medications has become a common issue because of their overuse. Therefore, plant-based therapies have piqued the interest of experts as a potential therapy for fungal infections [5]. The plant world has long been a hotbed for numerous natural chemicals with unique structures, which has piqued researchers’ curiosity in studying various plant species to this day. Numerous studies have shown that plants are a source of bioactive secondary metabolites, such as alkaloids, terpenoids, and saponins, all of which have demonstrated antifungal activities [89]. Traditional systems of medicine have also reported that medicinal herbs are fruitful candidates for treating animal and human mycoses. Furthermore, they can be used to develop novel antifungal drugs [88]. Accordingly, this section aims to explain the current situation regarding *T. aphylla* and its antifungal compounds, which may help to develop more effective antifungal medicines in the future.

A study was conducted to determine the effectiveness of ethanolic extracts of *T. aphylla* (2000 ppm, 1000 ppm, and 500 ppm) against six pathogenic fungi: *Fusarium oxysporum, Aspergillus niger, Aspergillus fumigatus, Aspergillus flavus, Saccharomyces cerevisiae,* and *Penicillium notatum*. Five different solvents (methanol, ethanol, acetone, chloroform, and distilled water) were used. The percentage suppression of fungus growth was shown to be dose-dependent. Terbinafine (synthetic drug) was administered in typical dosages with distilled water to test its effect against the various fungi. Terbinafine constrained the increase in *A. flavus, A. fumigatus, A. niger, F. oxysporum, P. notatum*, and *S. cerevisiae* at concentrations of 65 ± 0.58, 72 ± 1.00, 70 ± 1.15, 59 ± 1.00, 60 ± 0.58, and 80 ± 0.58 (µg/mL of PDA medium), respectively. The most effective solvent was chloroform, which prevented 97.68% ± 0.58% growth of *F. oxysporum*, 9.37% ± 0.33% growth of *A. niger*, 92.68% ± 3.33% growth of *S. cerevisiae*, 91.46% ± 2.08% growth of *A. fumigatus*, 88.48% ± 0.88% growth of *A. flavus*, and 87.95% ± 1.15% growth of *P. notatum*. Outcomes were statistically correlated to a negative control group, and most effects were highly significant (*p* ≤ 0.000). The stem–bark extract of *T. aphylla* showed the highest percentage of suppression with chloroform, followed by ethanol, acetone, methanol, and distilled water [90]. The methanolic extract of the bark of *T. aphylla* was examined in a study to counter bacterial pathogens in vitro to validate as *Staphylococcus aureus*, *Bacillus subtilis, Escherichia coli, and Salmonella typhi*, and fungal strains *Candida albicans* and *Aspergillus flavus* were tested using the disc diffusion assay with 25, 50, 75, and 100 mg/mL stock solutions prepared in dimethyl sulfoxide. The extract’s inhibitory impact in millimeters was calculated by measuring the zone of growth inhibition surrounding the discs and comparing it to the reference medication. *T. aphylla* bark established a marked zone of inhibition at higher concentration for most fungal and bacterial strains. According to the findings, *T. aphylla* produces bioactive and phytochemical substances used in healthcare [81]. However, there is a scarcity of research and comprehensive reviews on *T. aphylla* as an alternative to antifungal medications. There is a need to study the structure–activity relationship, molecular mechanism, and potential synergistic effects of components of *T. aphylla*. These findings suggest the need for further research on *T. aphylla* to manage antifungal diseases.

### 5.5. Periodontal Disease

After dental caries, periodontitis is the second most prevalent dental disease worldwide [91,92]. It is caused by forming an organized biofilm of bacterial strain, so-called dental plaque, in the subgingival area, affixed to host tissues and cells. This ultimately results in loss of supporting tooth structure [93,94]. The most common cause of periodontal diseases is infection and inflammation of the alveolar bone and gingiva, which supports the tooth in the socket [95]. Gingivitis is a disease of the gums in which they become red and swollen with a high bleed tendency without loss of supporting tooth structure [96,97,98]. Adults are more likely to get periodontal disease, mainly slow and moderately progressive periodontitis. Biofilms are multispecies bacterial colonies that invade the oral cavity as plaque, causing dental cavities and periodontal disease [99]. In a study, three herbs were examined to determine the effectiveness against pinpointed dental biofilm-forming bacteria. Pathogenic strains that form dental biofilm were isolated, grown, and evaluated using PCR-based 16S ribosomal RNA (or 16S rRNA) nucleotide sequences [27].

**Figure 5:**
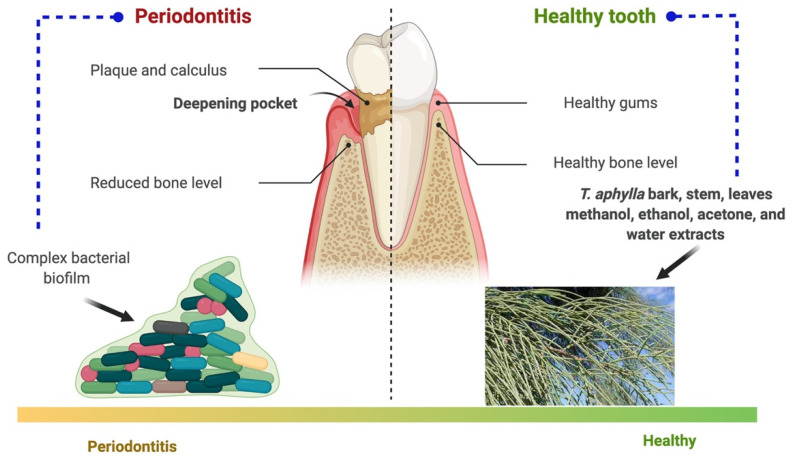
Periodontal disease and the probable role of *T. aphylla*.

*T. aphylla*, *Melia azadirachta*, and *Acacia arabica* were the three crucial medicinal plants investigated to determine their antibacterial activity against biofilm-forming strains. The findings showed that extracts of these three medicinal plants can be utilized against pathogenic bacteria that cause dental biofilms. Phylogenetic examinations explored 19 strains linked to Firmicutes, Actinobacteria, and Proteobacteria. Eleven of the 19 isolates were discovered to have a strong biofilm-forming potential, and an antibiotic activity assay revealed that the chosen herbs suppressed their development. Therefore, the findings showed that extracts of these medicinal plants can be utilized to guard against pathogenic bacteria that cause dental biofilms. Therefore, pharmaceutical companies should include them in dental care products [27]. Periodontal disease and the probable role of *T. aphylla* are illustrated in Figure 5.

### 5.6. Anticholinesterase Activity

Anticholinesterases are a class of medicines that inhibit acetylcholinesterase from destroying the neurotransmitter acetylcholine in the nervous system. Acetylcholine is a neurotransmitter that operates in the parasympathetic nervous system. It is part of the autonomic nervous system due to which smooth muscle contraction, secretion, and blood vessel dilation occur. Anticholinesterase prevents acetylcholine from being destroyed, allowing elevated levels of the neurotransmitter to accumulate at the spots of the mechanism. As a result, it provokes the parasympathetic nervous system, drops the heart rate, decreases blood pressure, stimulates secretion, and activates smooth muscle compression. The most beneficial application of anticholinesterase as a family of medications is in the treatment of Alzheimer’s disease, where decreased acetylcholine transmission contributes to the illness’s neuropathology [100].

Major constituents of *T. aphylla* include polyphenols, triterpenes, tamarixellagic acid, and dehydrotrigallic and dehydrodiagllic acids [12,101]; they have a potential role in preventing chronic and degenerative diseases. Traditional medicine uses *Tamarix* species as a stimulant of perspiration, an astringent, and a diuretic. The biological activities of several *T. aphylla* extracts were investigated in comparative research. At the measured concentration of 1110.00 g/mL and under the assay conditions, inhibitory percentages of 21.00%, 11.20%, and 9.16% were established for AcOEt, EtOH, and MeOH leaf extracts, respectively, with modest activity against AChE. Stem extracts were prepared using the same solvents and revealed inhibitory percentages of 16.19% (AcOEt), 17.45% (EtOH), and 10.20% (MeOH). In the case of butyrylcholinesterase (BuChE), *T. aphylla* extracts were ineffective in terms of inhibition. When it came to inhibiting AChE and BuChE, the examined extracts were less effective than the positive control galantamine, which was evaluated under identical circumstances (IC_50_ = 1.95 and 4.71 μg/mL, respectively). The absence of action against BuChE and the modest activity of the studied extracts against AChE can be explained by the potential antagonism of physiologically active *p*-coumaric acid (only detected in the AcOEt extract), with high anti-ChE activity against both BuChE and AChE [102]. There are many other unidentified compounds, but polar MeOH and EtOH extracts are rich in ellagic and gallic acids, which have been proclaimed as effective blockers of AChE [46]. Mahfoudhi et al. exhibited that activity toward superoxide anion radicals (O2^−^), DPPH radicals, and nitric oxide radicals (•NO) was accomplished in a concentration-dependent mode. *T. aphylla* also had modest anti-acetylcholinesterase activity but no anti-butyrylcholinesterase effects. MeOH extracts had IC_50_ values ranging from 8.41 to 24.81 g/mL against α-glucosidase [54].

### 5.7. Analgesic Activity

*Pain* is an unpleasant sensation because of actual or prospective tissue injury. The experience of pain is produced in the early stages via direct activation of sensory nerve fibers. Contrastingly, inflammatory mediators are the main cause of trouble in the later stages. Contemporary anti-inflammatory and antipain medications are helpful, but they have serious side-effects including anemia, ulcers, endocrine disruption, and osteoporosis. On the other hand, numerous prospective phytoconstituents from herbs have shown analgesic activities; therefore, people are more interested in medicinal plants than synthetic medicines. In addition, these plants generally have adequate biological activity with fewer adverse effects [103].

In this section, we review the analgesis activity of *T. aphylla*. The findings of this section allow identifying *T. aphylla* and its secondary metabolites, which can be helpful in clinically advanced herbal drugs as analgesics with fewer adverse effects and improved bioavailability. One study examined the analgesic effect of an aqueous ethanol (70:30) extract of *T. aphylla* aerial parts prepared using cold maceration. The study investigated analgesic efficacy in mice using eddy’s hot plate method, formalin-induced paw licking, and the acetic acid-induced writhing. When compared to the normal control, the aqueous ethanol extract of *T. aphylla* inhibited 42% (*p* < 0.005) of acetic acid-induced writhing, 63% (*p* < 0.005) of formalin-induced paw licking, and 42% (*p* < 0.005) of response time. Outcomes of the examination showed that the aqueous ethanolic extract of *T. aphylla* has promising analgesic properties [21]. The results of this study encourage the application of *T. aphylla* to alleviate fever and pain. Moreover, more examinations are required to establish the cellular mechanisms and classify the constituents of structurally effective substances for standardization. Another study was conducted in Khartoum, Sudan, for 6 months. The Phyto-342 components of *T. aphylla* were detected using standard techniques from the laboratory sheet. Animal models specified in conventional methods evaluated the percentage of granuloma tissue, inhibition percentage of edema, stabilizing ability of membrane, and analgesic effectiveness. In addition, 100 mg/kg and 200 mg/kg dose regimens of *T. aphylla* considerably enhanced the reaction time and lowered the body temperature (*p* < 0.05) of rats compared to acetylsalicylic acid. Furthermore, the ethanolic extract of *T. aphylla* has shown membrane-stabilizing and analgesic properties [104].

### 5.8. Antidiabetic Activity

According to the WHO, 220 million humans are affected by diabetes; this number is anticipated to be double by 2030 [105]. However, glucose is an important energy source for the cells that make up tissues and muscles, and it fuels the brain [106,107,108]. How the body uses glucose (blood sugar) is the cause of various diseases. The state of hyperglycemia is called diabetes mellitus [109]. This ailment hits the cardiovascular system, nervous system, kidneys, and eyes [110]. Inflammation is due to the triggering of various immune cells leading to the death of pancreatic beta cells death via several inflammatory cytokines, thus resulting in insulin resistance [111]. Reactive oxygen species and oxidative stress are major causes of diabetes [112]. Diabetes is a severe metabolic condition that is treated using a variety of medicinal herbs in traditional medicine [113]. The main objective of all diabetes care is to keep blood glucose levels stable. Traditional medicine and natural products have the potential for developing therapeutics against chronic inflammatory diseases. Many drugs are obtained from these herbs with limited adverse effects or no side-effects [64]. They are efficient herbal medications and natural antioxidants because they include antidiabetic chemicals, including alkaloids, phenolics, tannins, and flavonoids, reducing glucose absorption in the intestine or improving pancreatic tissue performance by boosting its performance in terms of insulin production [113]. This portion of the review aims to gather existing knowledge on *T. aphylla* regarding its antidiabetic potential with future research directions.

*T. aphylla* is used as a carminative, diuretic, and anti-inflammatory herbal remedy. A study was carried out to investigate the antihyperglycemic activity of *T. aphylla* leaves in STZ-NIC-stimulated diabetes in Wistar Albino rats. *T. aphylla*’s methanol extract was examined to determine its acute toxicity. Different doses (100 mg, 250 mg, and 400 mg/kg body weight per day) of *T. aphylla* leaves extract were administered for 21 days to diabetic Wistar rats. Multiple parameters were examined, such as hemoglobin, glycosylated hemoglobin, and fasting blood glucose levels. A total of 50 male Wistar rats, aged 8–10 weeks and weighing 220–320 g, participated in this examination. All rats stayed in an airy room with a humidity of 40–50% and a temperature of 25 ± 2 °C in a 12 h light/dark cycle. For 2 weeks before the experiment and throughout the experiment, rats were fed regular laboratory food and had an available water supply. Rats were fasted for 12 h before receiving a diabetic injection. A single intraperitoneal (*i.p.*) injection of STZ (65 mg/kg body weight (b.w.)) freshly dissolved in ice-cold saline was used to induce diabetes [114]. An equal volume of normal saline was injected into control animals. Nicotinamide (180 mg/kg, *i.p.*) was dissolved in normal saline and given 15 min before STZ administration [115]. High pancreatic insulin release could be the cause of deadly hypoglycemia. At the end of STZ treatment over the next 24 h, a 10% glucose solution was fed to rats to subdue drug stimulated hypoglycemia [7]. In an acute toxicity investigation, animals given a methanol extract of *T. aphylla* showed no change in their behavior pattern [8]. Male Wistar mice were used in the experiment [116]. There was no evidence of death in the extract-treated mice, and their performance looked to be normal. Up to the 1500 mg/kg dosage, no mortality or toxic response was observed until the end of the day. Blood glucose levels were assessed in normal control and experimental animals on days 1, 3, 7, 14, and 21 of the therapy. Compared to normal rats, STZ-NIC-stimulated diabetic rats exhibited a considerable rise in blood glucose levels. In the analysis of diabetic control, *i.p.* management of *T. aphylla* (400 mg/kg body weight) substantially reduced FBS (fasting blood sugar). The 21 days of therapy using *T. aphylla* were examined in terms of glycosylated hemoglobin and hemoglobin levels in usual and STZ-stimulated diabetic rats. Analysis of normal control and diabetic rats showed a substantial drop in hemoglobin and a rise in HbA1c. In contrast to diabetic control rats, *i.p. T. aphylla* improved hemoglobin and lowered HbA1c. Compared to the normal control rats, STZ treatment led to changes in HbA1c, hemoglobin, and blood glucose. The results showed that *T. aphylla* leaf extract lowers blood glucose levels in people with diabetes. Furthermore, it can avoid diabetes complications caused by high blood glucose levels [8].

Antidiabetic properties are linked to the presence of terpenoids, flavonoids, coumarins, and a host of different secondary plant metabolites of *T. aphylla* [117]. Galls of *T. aphylla* comprise polyphenols, such as gallic acid, ellagic acid, isoferulic acid, dehydrodigallic acid, and juglanin, as well as flavonoids such as isoquercitrin, quercetin glucoside, and its methyl derivatives tamarixetin and tamarixin [4]. Gallic acid (GA) is a phytochemical with several biological functions, and it offers antioxidant and anti-advanced glycation properties. It also inhibits angiotensin-converting enzyme [118]. Another study examined the combined effect of metformin with secondary metabolites such as gallic acid in improving antioxidant activity and glucose metabolism in diabetic rats. The pancreas and liver of diabetic rats were examined after administering metformin and GA. Outcomes showed that metformin and GA decreased the levels of IL-6 and TNF-α, and the expression of ATF4. Results of the study validated that the combination of GA and metformin is more effective than metformin alone [119]. Furthermore, another study validated that andrographolide and gallic acid synergistically reduce the level of blood glucose [120]. Therefore, GA meets the criteria for being investigated as a therapeutic candidate in diabetic nephropathy, one of the most severe complications of diabetes. GA therapy significantly increased albumin and protein levels while reducing plasma creatinine and blood urea nitrogen levels. GA also enhanced creatinine clearance. According to oxidative stress markers, GA treatment in mice exhibited significantly lessened oxidative stress in the kidney. In diabetic nephropathy, it also lowered TGF-β_1_ levels in the blood and renal tissue. According to the findings, *T. aphylla* is a good source of GA, which can be utilized successfully in treating diabetic nephropathy [118].

A flower extract of *T. aphylla* yielded tamarixetin 3,3′-disodium sulfate and glycosylated isoferulic acid [18]. An examination was carried out to determine the free-radical scavenging and antiglycation properties of isoferulic acid (IFA). IFA’s free-radical scavenging activity was calculated employing DPPH, FRAP, and metal adsorption assays. Various spectroscopic techniques were used to measure the antiglycation activity of IFA. The research outcomes displayed the effect of IFA on ROS and glycation via a structural transformation of HAS (human serum albumin). A comparison of the antioxidant properties of IFA with GA, employing many biochemical and biophysical methods, showed that IFA has ideal properties [121].

**Figure 6:**
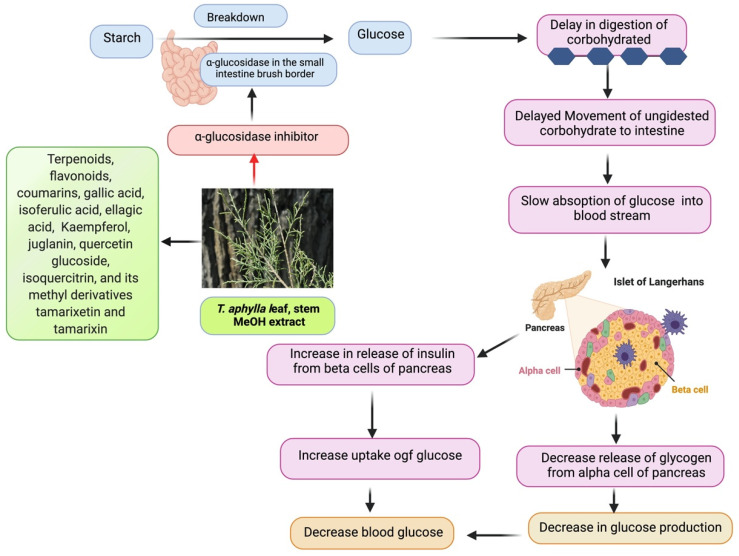
The proposed mechanism underlying the glucosidase-inhibitory properties of *T. aphylla*.

There is a constant rise in blood glucose in type 2 DM due to the breakdown of starchy foods by glucose absorption and α-amylase in the small intestine by α-glucosidase [122]. Suppression of α-glucosidase activity has been linked to a reduction in plasma glucose levels and suppression of postprandial hyperglycemia. Consequently, it represents a successful remedial approach to treat type 2 DM by lowering glucose levels in the blood [123]. In contrast, α-glucosidase blockers, such as voglibose, acarbose, and miglitol, lead to gastrointestinal adverse effects such as diarrhea and flatulence. As a result, it is critical to discover indigenous natural resources free of or with fewer adverse effects [124]. A study was carried out by Mahfoudhi et al. in which MeOH extracts from the leaves and stems of *T. aphylla* were examined to inhibit α-glucosidase actions. *T. aphylla* inhibited α-glucosidase in a concentration-dependent manner, with 91.98% and 88.73% inhibition at concentrations of 113.64 and 28.41 g/mL, respectively. In this study, IC_50_ values were found for leaves (24.81 μg/mL) and stem (8.41 μg/mL). These results were lower than those reported for acarbose (IC_50_ = 300 g/mL), the positive control. The inhibition by *T. aphylla* extracts was significantly higher compared to other botanicals employed in diabetic medication, such as *Glycyrrhiza uralensis* (IC50 = 20.1 mg/mL) [125] and *Viscum album* (IC_50_ = 11.7 mg/mL) [54,126]. It has been reported in previous studies that quercetin, ellagic acid, and kaempferol inhibited α-glucosidase [127,128]. Therefore, it can be concluded that *T. aphylla*’s MeOH extracts exhibited higher antidiabetic properties due to the presence of quercetin, ellagic acid, and kaempferol. It can also be concluded that α-glucosidase displays suppressive properties, whereas *T. aphylla* exhibits antioxidant properties. As a result, *T. aphylla* might be beneficial in the therapy of DM. Furthermore, *T. aphylla* works by blocking a pathology-related enzyme and strengthening the patients’ antiradical defenses [54]. The proposed mechanism underlying the glucosidase-inhibitory properties of *T. aphylla* is shown in Figure 6.

Separating the active components of *T. aphylla* and the molecular interactions of its constituents for investigation of their therapeutic effects requires more research. As a result, we must keep looking for new and, if possible, more effective antidiabetic properties of *T. aphylla*, and its vast reservoirs of phytoconstituents might represent an excellent alternative. Despite this, there is little research on the plant’s antidiabetic properties.

### 5.9. Cytotoxic Activity

Natural materials have been recognized as possible sources of human medicines since ancient times. Over 50% of all pharmaceutical drugs are still natural ingredients [129,130]. Currently, natural sources such as plants, microorganisms, and sea creatures account for more than 60% of commercially accessible anticancer medicines [131,132,133,134]. A total of 3000 plant species have been utilized to treat various kinds of cancer [132,135]. Every year, many chemical entities are introduced to the market or undergo clinical studies [129]. Plant bioactive compounds and phytochemicals can prevent more than 20% of cancer diagnoses and 200,000 cancer-related deaths per year. Several plants are being studied for cancer prevention due to their lesser toxicity, safety, and antioxidant qualities [80,132,136]. Since the 1940s, natural products or derivatives of natural products have accounted for 48.6% of the 175 small compounds authorized for cancer treatment [137]. Therefore, it is essential to obtain valuable and harmless unique agents with cytotoxic properties with minimal or no adverse effects on normal cells, along with extraordinary potency at various sites, as well as low cost and acceptance in the community. Consequently, conducting various in vitro, in vivo, and clinical examinations is also necessary [138].

**Figure 7:**
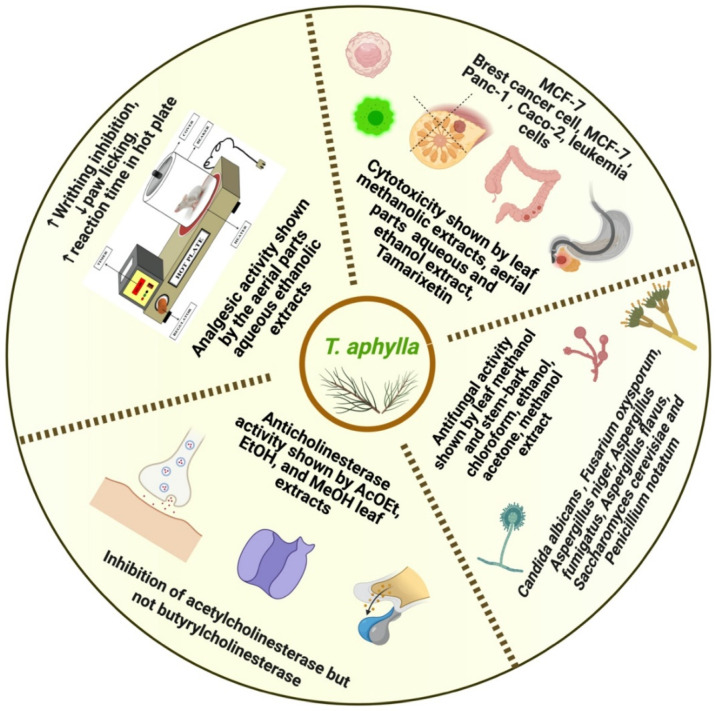
Analgesic, anticholinesterase, cytotoxicity, and antifungal activity of *T. aphylla*.

It is essential to test cytotoxicity to establish the safety profile of *T. aphylla* and obtain active constituents [139]. To investigate the cytotoxic impact, fresh and disease-free leaves of *T. aphylla* were collected from several geographical sites in Saudi Arabia. In vitro, the anticancer and cytotoxic effects of *T. aphylla* leaves were examined, employing MCF (breast adenocarcinoma cells) as carcinoma cells and Vero cells as normal cells. The 50% cytotoxicity concentration denoting the concentration leading to a 50% reduction in cell viability was investigated. The result of the MTC colorimetric test was determined to be 2000 g/mL. A mild cytotoxic effect was observed at high concentrations of leaf extract of *T. aphylla* on the Vero cell line with a 50% cytotoxicity concentration >100 g/mL. In a concentration-dependent manner, the methanolic extract inhibited MCF cancer cells. The findings of the cytotoxicity test of *T. aphylla* leaf methanolic extracts on Vero cells indicated that this plant is potentially nontoxic. As a result, the findings of this investigation suggested that *T. aphylla*’s cytotoxic strength should be considered a potential applicant for future testing in vivo and in vitro against various additional cell types. This is in line with earlier research on the intriguing anticancer properties of *Tamarix* species [80]. Antiproliferative effectiveness was found against MCF-7 cells using the leaf extract of *T. aphylla*; thus, it should be employed in future antiproliferative studies. GC–MS and GC methods were used to examine the antiproliferative activity of the essential oil composition of aerial parts of *T. aphylla* aqueous extract (AE) and ethanol extract (EE), as well as to investigate potential cytotoxicity against pancreatic carcinoma, colorectal adenocarcinoma, human breast adenocarcinoma (MCF-7), and normal human fibroblasts. It was found in the GC–MS analysis of *T. aphylla* EO that the principal component was 6,10,14-trimethyl-2-pentadecanone in terms of predominance and richness among non-terpenoid nonaromatic hydrocarbons (52.39%). Biological results showed that *T. aphylla* extracts exhibit cytotoxicity in a dose-dependent manner against most tested cell lines. Nevertheless, potent effects were recorded against MCF-7 cells with IC_50_ values of 2.17 ± 0.10 and 26.65 ± 3.09 μg/mL for *T. aphylla* EE and AE, respectively. *T. aphylla* AE has exhibited a comparable cytotoxic profile to control drug cisplatin (IC_50_ value of 1.17 ± 0.13 μg/mL), along with a complete safety profile against normal fibroblast cells (IC_50_ of *T. aphylla* AE versus cisplatin: 79.99 ± 4.90 versus 9.08 ± 0.29 μg/mL). In addition, at concentrations less than 30 g/mL, the AE showed no antiproliferative potential against Panc-1 or Caco-2 cancer cell lines. This study concluded that *T. aphylla* extracts might be cytotoxic agents with selective cytotoxicity profiles and high safety [140]. Another study used the brine shrimp method to examine the cytotoxic property of methanol extracts of *T. aphylla* leaves. This method showed a 70% mortality rate to *T. aphylla* extract at a 500 μg/mL concentration [141]. The cytotoxic potential of *T. aphylla* bark extract against human erythrocytes was assessed. *T. aphylla* bark extract exhibited 3.48 ± 1.08 hemolytic activity compared to the positive control. The studied *T. aphylla* extracts had a lower hemolytic potential, making them safe in pharmaceutical applications [142]. Many studies reported a high content of polyphenols, tannins, and flavonoids [143,144,145]. Various phenolic compounds isolated from Tamaricaceae were tested against different cancer cell lines for their cytotoxic effects [146]. Tamarixetin was discovered to be cytotoxic to leukemia cells [147]. In a time- and dose-dependent manner, this compound suppressed proliferation, caused apoptosis, and stopped cell-cycle progression [148]. Ellagitannins have also exhibited anticancer and antiangiogenic properties mediated by the host [149]. As shown by TLC tests, secondary metabolites and phenolic fractions may account for the higher cytotoxic property found in various studies of the extracts of *T. aphylla* [140]. *T. aphylla* extracts have shown antiproliferative activities against MCF-7 cancer cell lines, which could be the basis to attract future research to develop novel cancer therapies. The reported analgesic activity mechanism, anticholinesterase action, cytotoxicity against different cell lines, and antifungal activity against different fungal strains of *T. aphylla* are shown in Figure 7.

## 6. Toxicity

Natural medicinal plants are important because they are rich in diverse phytochemical components. In addition to being extensively accessible and commercially useful, these phytochemical substances have pharmacological effects in treating various medical situations. Plant phytochemical substances have been investigated in numerous experiments to discover the molecular basis of their interaction with medicines and illnesses [150]. However, plants are potentially toxic due to their chemical components; as a result, certain plants employed in traditional medicine are intrinsically harmful [151,152,153]. *T. aphylla* (L. Karst) methanol extract was examined for acute toxicity. In the three test groups, a leaf extract of *T. aphylla* was administered intraperitoneally with doses of 100 mg, 250 mg, and 400 mg/kg body weight to the mice of every group per day. The control group was administered saline; mice were examined in terms of the following factors for 2 h: (a) behavioral determinants: agitation, alertness, dread, and irritability; (b) neurological determinants: reaction, impulsive action, touch reaction, gait, and pain response; (c) autonomic determinants: urination and excretion. They were monitored for mortality or lethality after 24 h. In an acute toxicity investigation, the methanol extract of *T. aphylla*-treated mice did not lead to changes in behavioral pattern. Male Wistar mice were used in the experiment. There was no evidence of death in the extract-treated mice, and their performance looked to be normal. No toxicity or mortality was observed at the end of the day after administering 1500 mg/kg; therefore, *T. aphylla’s* extracts are nontoxic [8]. The results of another toxicological study on *T. phylla* extract at various doses up to 2000 mg/kg did not show any adverse effect [70]. Another study of *T. aphylla* extract showed a protective effect against DOX cardiotoxicity [154]. Toxicity was negligible in *T. aphylla* from Saudi Arabia and Pakistan [144]. Table 3 presents the results of various preclinical studies.

## 7. Concluding Remarks

This review covered the recent literature related to the ethnobotanical uses, phytochemical compounds, pharmacological effects, and toxicology of *T*. *aphylla.* The findings of this work establish the connection between *T. aphylla* in the traditional medication system and its impact on other biological properties such as antibacterial, anti-inflammatory, antidiabetic, analgesic, wound healing, antifungal, and anticholinesterase activities. The leaves and stem/bark seem to be the most effective plant parts of *T. aphylla* as these parts showed several therapeutic activities. However, there is a need to study the isolation and characterization of phytoconstituents, as well as the molecular mechanism of components from these plant parts of *T. aphylla*. Different chemical compounds such as polyphenols, flavonoids, and tannins account for the various biological activities of *T. aphylla*. Isolation of secondary metabolites and their examinations in vitro and in vivo to test various pharmacological activities could attract future research and drug discovery.

However, nonclinical studies have shown that the *T. aphylla* has significant antimicrobial, antioxidant, and cytotoxic activities. Thus, more extraction, isolation, and purification studies are needed to develop potential drugs to treat various ailments. The findings of many studies exhibited that the leaf extract of *T. aphylla* is nontoxic with no reported mortality, while toxicity of *T. aphylla* was found to be negligible in studies from Saudi Arabia and Pakistan. The pharmacological properties of *T. aphylla*, according to study findings, support its traditional applications. Clinical trials establishing the health benefits were not found; therefore, high-quality preclinical studies and well-designed clinical studies are needed to confirm the efficacy and safety of *T. aphylla* in people.

Moreover, herbal supplements have not received the same scientific scrutiny and are not as strictly regulated as medications. Natural products are regulated by the US Food and Drug Administration (FDA), but not as strictly as prescription or over-the-counter (OTC) drugs. Natural products should be free of contaminants and must meet specific quality standards. Some herbs can cause serious side-effects when mixed with aspirin, blood thinners, and blood pressure medications. Many herbal supplements can affect the success of surgery. Some may decrease the effectiveness of anesthesia or cause dangerous complications, such as bleeding. Few natural products have been tested in children or have established safe doses. Moreover, older adults may metabolize medications differently. Therefore, it is essential to learn about the potential benefits and side-effects of herbal supplements before they are prescribed in the clinic.

## Figures and Tables

**Table 1:** *T. aphylla’s* ethnomedicinal usage in many places around the world.

Geographical Location	Parts of the Plant Used	Indication	Route of Administration	Results	References
Southeastern Morocco	Leaf	Hypertension	S/Decoction	Information was collected from the respondents of Errachidia province regarding the plants used for hypertension, one of which was *T. aphylla*.	[34]
The central region of Abyan governorate, Yemen	Bark and leaf	Abdominal pain, hair loss, cough, and asthma	S, Lo/Infusion, decoction	Ethnobotanical survey of medicinal plants showed that residents of Yemen use *T. aphylla* for abdominal pain, hair loss, cough, and asthma.	[35]
Northwestern part of Pakistan	Whole plant	Abscesses and wounds, rheumatism, jaundice, bad evils	S, Lo/Decoction of ash, ash, boiled leaves	*T. aphylla* is one of the plants enlisted in the Holy Quran, Islamic literature, and Ahadith with ethnobotanical relevance.	[20]
District Sargodha, Punjab, Pakistan	Bark	Measles, aphrodisiac	S/Powdered with oil, smoke	*T. aphylla* is one of the most used timber species in district Sargodha.	[36]
Peshawar Valley of Pakistan	Bark and leaf	Paralysis, abdominal pain, tetanus, rheumatism, and wound healing	S, Lo/Powder extract	Ethnobotanical study on medicinal plants exhibited that residents of Peshawar use *T. aphylla* in various diseases.	[37]
District Karak, Pakistan	Leaf	Animal pain killer for wounds, in bird flu	S/Smoke	This study showed the ethnoveterinary use of *T. aphylla*.	[38]
Pakistan	Fruit	Diabetes	S/Decoction	*T. aphylla* can be used as antidiabetic medication.	[39]
Karamar Valley, Swabi, Pakistan	Bark	Jaundice, rheumatism, infection of gums and teeth	ND	*T. aphylla*’s extract showed activity against the biofilm-causing bacteria in periodontal diseases.	[27]
Chenab riverine area, Punjab province, Pakistan	Leaf and bark	Cough and cold, eye infection, wounds and boils, febricity	S, Lo/ Poultice, paste, decoction, ash	Local people employ *T. aphylla* to cure various ailments in numerous regions of Pakistan.	[26]
Rajhan Pur, Punjab, Pakistan	Root, leaf	All contagious diseases, jaundice, smallpox, leprosy, tuberculosis	ND	A field survey showed various uses of *T. aphylla* in multiple diseases.	[28]
Central Sahara	Shoots	Aid to menstruation, postpartum care, fever	S/Decoction	Results showed that people traditionally use *T. aphylla* as a potential traditional healer.	[40]
Jordan, North Badia	Leaf	Fever	S/Decoction	*T. aphylla* is an effective medication in pain and inflammations.	[41]

Abbreviations: T: *Tamarix*, L: leaf, B: bark, F: fruit, G: gall, R: root, SH: shoots, WP: whole plant, S: systemic, Lo: local, ND: not defined.

**Table 2:** Some of the primary and secondary metabolites of *T. aphylla*.

Phytochemical Category	Phytochemical Name	Structure	Part/Extract	References
Aromatic hydrocarbon Carbohydrate	Fructose	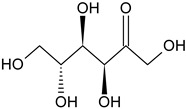	G, Gu/EtAc	[53]
	Glucose	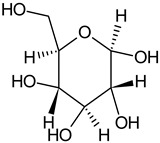	G, Gu/EtAc	[53]
	Raffinose	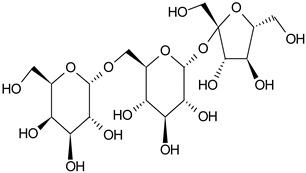	G/EtAc	[53]
	Ribose	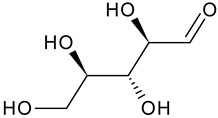	G/EtAc	[53]
	Sucrose	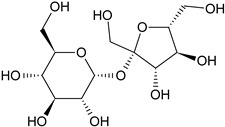	G/EtAc	[53]
	Xylose	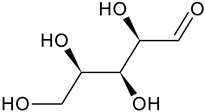	G, Gu/EtAc	[53]
Flavonoids	Apigenin	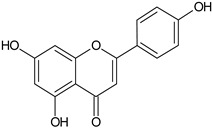	SS, L, B, EtOH	[54]
	Isoquercetin	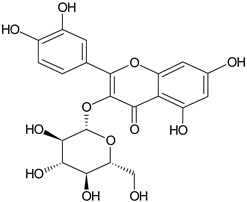	G/EtAc	[53]
	Isorhamnetin	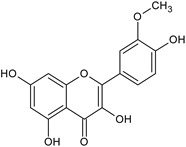	L, S, B/EtOH, aqueous	[17,55]
	Juglanin	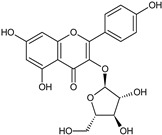	G/EtAc	[53]
	Kaempferide	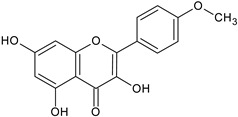	Ap/EtOH	[17,55,56]
	Kaempferol	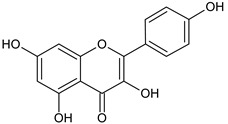	L and S/MeOH, EtOH	[17,55]
	Luteolin	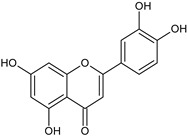	L and S, EtOH	[17]
	Quercetin	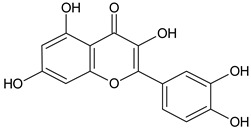	SS, L/EtOH	[17,56]
	Quercetin dimethyl ether	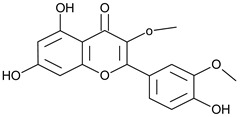	L/Aqueous	[55]
	Quercetin-3-rhamnoside	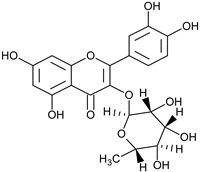	F/EtOH	[57]
	Quercetin 3-*O*-galactoside	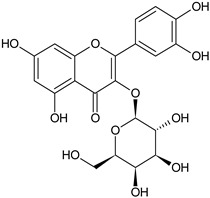	L and S/MeOH, EtOH	[54]
Phenolic acid	Caffeic acid	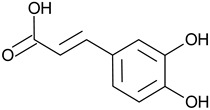	L and S, EtOH	[17]
	Dehydrodigallic acid	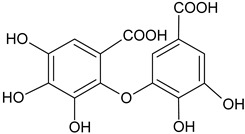	G, EtAc	[53]
	Dehydrotrigallic acid	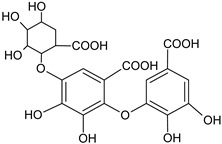	G/aqueous ethanolic acid	[49]
	Ellagic acid	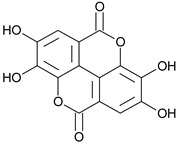	G, debarked heartwood	[53]
	Gallic acid	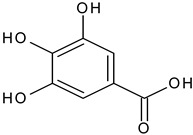	L, debarked heartwood	[55]
	Isoferulic acid	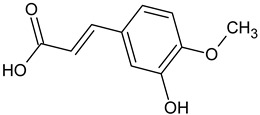	Debarked heartwood, G, EtAc	[53]
	*p*-Coumaric acid	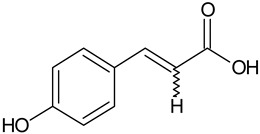	L and S/EtOH, aqueous	[17,55]
	Syringic acid	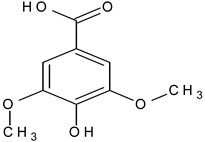	L, debarked heartwood/aqueous	[55]
Phenolic glycoside	Dehydrodigallic-xanthone	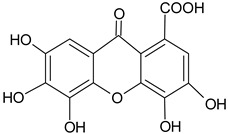	G/Aq-EtOH	[49]
	Dehydrotrigallic-xanthone	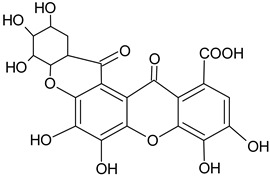	G/Aq-EtOH	[49]

Abbreviations: G: gall, L: leaf, S: stem, F: flower, B: bark, Sh: shoot, Ap: aerial parts, Pet: petroleum, EO: essential oil, Gu: gum, R: root, Aq: aqueous, EtOH: ethanol, MeOH: methanol, EtAc: ethyl acetate.

**Table 3:** Characteristics of included preclinical studies.

Pharmacological Activity	Plant Part	Solvent for Extraction	Model/Induction/Agent/ Species	Dosage or Concentration	Sample Size/Pathogens	Results	References
Antidiabetic	Leaf	MeOH	In vivo study, diabetic Wistar rats (induced by nicotinamide + STZ)	100, 250, 400 mg/kg/ day	50 male Wistar rats	Outcomes showed nontoxic and antidiabetic properties	[8]
Antidiabetic	Leaf, stem	MeOH	In vitro study, α-glucosidase inhibitory assay	1–1000 μg/mL	-	Antidiabetic properties were shown	[54]
Antipyretic, analgesic, and anti-inflammatory properties	Aerial parts	Aqueous ethanolic extract	In vivo, edema and yeast-induced, carrageenan-induced paw edema	100 mg/kg/day	Swiss albino male mice (20–30 g), three groups of six mice	Outcomes revealed antipyretic and analgesic activities with lessened anti-inflammatory action	[21]
Anti-inflammatory and wound healing	Leaf	EtOH	In vivo, carrageenan-induced paw edema	15%, 25% in gel base	Wistar rats (180–200 g)	Outcomes showed gel usefulness	[70]
Antimicrobial	Leaf, stem, bark	Diverse extracts	In vitro study, micro-titer assay, agar well diffusion method	1 mg/mL	-	Helpful against 11 biofilm-forming strains	[27]
	Leaf	MeOH extract	In vitro: antibacterial activity against *Escherichia coli* and *Staphylococcus aureus*, agar dilution method against three *Aspergillus* species	0.86–30 mg/ mL	-	No considerable antibacterial effect	[141]
Antifungal	Leaf	MeOH extract	In vitro: dilution of agar tubes	67 μL (200 μg/mL)	-	*T. aphylla* showed significant antifungal activity: 70%, 55%, and 54% against *Aspergillus flavius*, *Aspergillus niger*, and *Aspergillus fumigatus,* respectively	[141]
	Stem, bark	Crude ethanolic extracts	In vitro	Concentrations 500 ppm, 1000 ppm, and 2000 ppm	Six pathogenic fungi	Maximum inhibition has been shown by *T. aphylla*	[90]
	Bark	MeOH	In vitro: disc diffusion assay	25, 50, 75, and 100 mg/mL	*Aspergillus flavus* and *Candida albicans*	*T. aphylla* inhibited most fungal strains at higher concentrations	[81]
Toxicity	Leaf	MeOH	In vivo, toxicity study in mice	500–2100 mg/kg/day	50 male, Wistar rats (220–320) g	Nontoxic	[8]
Toxicity	Leaf	EtOH	In vivo, toxicity study in rats	Doses administered: 50, 100, 300, 1000, and 2000 mg/kg body weight	Wistar rats	No toxicity was reported up to 2000 mg/kg body weight	[70]
Toxicity	Leaf	MeOH	In vivo	50–500 mg/ mL	Brine shrimp	Brine shrimp showed a 70% mortality rate	[141]
Wound healing	Leaf	EtOH	In vivo, excision wound and induced paw edema model	15%, 25% in gel base	Wistar rats	*T. aphylla* leaf extract possesses wound-healing properties	[70]
Periodontal disease	Bark, stem, leaves	Methanol, ethanol, acetone, and water extracts	In vitro, phylogenetic analysis	*-*	Pathogenic bacteria from dental biofilms.	*T. aphylla* showed potent antibiofilm activity	[27]

## References

[1] Narendhirakannan R.T., Subramanian S., Kandaswamy M. (2006). Biochemical evaluation of antidiabetogenic properties of some commonly used Indian plants on streptozotocin-induced diabetes in experimental rats. Clin. Exp. Pharmacol. Physiol..

[2] Hussain A., Ahmad M., Wahab S., Hussain M., Ali M. (2011). A review on pharmacological and phytochemical profile of Asparagus racemosus Willd. Pharmacologyonline.

[3] Alsayari A., Muhsinah A.B., Almaghaslah D., Annadurai S., Wahab S. (2021). Pharmacological efficacy of ginseng against respiratory tract infections. Molecules.

[4] Ali M., Alhazmi H.A., Ansari S.H., Hussain A., Ahmad S., Alam M.S., Ali M.S., El-Sharkawy K.A., Hakeem K.R. (2019). Tamarix aphylla (L.) Karst. Phytochemical and Bioactive Profile Compilations of Less Discussed but Effective Naturally Growing Saudi Plant. Plant and Human Health, Volume 3.

[5] Ahmad M.F., Ahmad F.A., Ashraf S.A., Saad H.H., Wahab S., Khan M.I., Ali M., Mohan S., Hakeem K.R., Athar M.T. (2021). An updated knowledge of Black seed (Nigella sativa Linn.): Review of phytochemical constituents and pharmacological properties. J. Herb. Med..

[6] Wahab S., Ahmad I., Irfan S., Siddiqua A., Usmani S., Ahmad M.P. (2021). Pharmacological Efficacy and Safety of Glycyrrhiza glabra in the treatment of respiratory tract infections. Mini-Rev. Med. Chem..

[7] Pradeepa S., Subramanian S., Kaviyarasan V. (2013). Biochemical evaluation of antidiabetic properties of Pithecellobium dulce fruits studied in streptozotocin induced experimental diabetic rats. Int. J. Herb. Med..

[8] Ullah R., Tariq S.A., Khan N., Sharif N., Ud Din Z., Mansoor K. (2017). Antihyperglycemic effect of methanol extract of Tamarix aphylla L. Karst (Saltcedar) in streptozocin–nicotinamide induced diabetic rats. Asian Pac. J. Trop. Biomed..

[9] Gaskin J.F. (2003). Tamaricaceae. Flowering Plants Dicotyledons.

[10] Baum B.R. (1978). The Genus Tamarix.—Jerusalem: The Israel Academy of Sciences and Humanities.

[11] Alnuqaydan A.M., Rah B. (2019). Tamarix articulata (T. articulata)—An Important Halophytic Medicinal Plant with Potential Pharmacological Properties. Curr. Pharm. Biotechnol..

[12] Bahramsoltani R., Kalkhorani M., Abbas Zaidi S.M., Farzaei M.H., Rahimi R. (2020). The genus Tamarix: Traditional uses, phytochemistry, and pharmacology. J. Ethnopharmacol..

[13] Samadi N., Ghaffari S.M., Akhani H. (2013). Meiotic behaviour, karyotype analyses and pollen viability in species of Tamarix (Tamaricaceae). Willdenowia.

[14] Mustafa Akhlaq A.M. (2011). New phenolic acids from the galls of *Tamarix aphylla* (L.) Karst. Int. Res. J. Pharm..

[15] Barakat H.H., Nada S.A. (1996). Chemical and Biological Investigations of the Constitutive Phenolics of Two Egyptian Folk-Medicinal Plants; A Novel Phenolic from the Galls of Tamarix aphylla. Nat. Prod. Sci..

[16] Nawwar M.A., Ayoub N.A., El-Rai M.A., Bassyouny F., Mostafa E.S., Al-Abd A.M., Harms M., Wende K., Lindequist U., Linscheid M.W. (2012). Cytotoxic ellagitannins from Reaumuria vermiculata. Fitoterapia.

[17] Mahfoudhi A., Prencipe F.P., Mighri Z., Pellati F. (2014). Metabolite profiling of polyphenols in the tunisian plant tamarix aphylla (L.) Karst. J. Pharm. Biomed. Anal..

[18] Nawwar M.A.M.M., Hussein S.A.M.M., Ayoub N.A., Hofmann K., Linscheid M., Harms M., Wende K., Lindequist U. (2009). Aphyllin, the first isoferulic acid glycoside and other phenolics from Tamarix aphylla flowers. ChemInform.

[19] Majumder P., Paridhavi M. (2013). A comprehensive ethno-phyto-pharmacological review on novel Indian medicinal plants used in polyherbal formulations. Int. J. Phytomed..

[20] Ahmad M., Zafar M., Sultana S. (2009). Salvadora persica, Tamarix aphylla and Zizyphus mauritiana-Three Woody Plant Species Mentioned in Holy Quran and Ahadith and Their Ethnobotanical Uses in North Western Part (D.I. Khan) of Pakistan. Pak. J. Nutr..

[21] Qadir M.I., Abbas K., Hamayun R., Ali M. (2014). Analgesic, anti-inflammatory and anti-pyretic activities of aqueous ethanolic extract of *Tamarix aphylla* L. (Saltcedar) in mice. Pak. J. Pharm. Sci..

[22] Laaroussi I. (2012). Natural Product Temporarily Tightening the Mucous Membranes of the Vagina. Patent.

[23] Marwat S.K., Rehman F.U., Khan M.A., Ahmad M., Zafar M., Ghulam S. (2011). Medicinal folk recipes used as traditional phytotherapies in district Dera Ismail Khan, KPK, Pakistan. Pak. J. Bot..

[24] Ullah S., Rashid Khan M., Ali Shah N., Afzal Shah S., Majid M., Asad Farooq M. (2014). Ethnomedicinal plant use value in the Lakki Marwat District of Pakistan. J. Ethnopharmacol..

[25] Zhang D., Yin L., Pan B. (2002). Biological and ecological characteristics ofTamarix L. and its effect on the ecological environment. Sci. China Ser. D Earth Sci..

[26] Umair M., Altaf M., Bussmann R.W., Abbasi A.M. (2019). Ethnomedicinal uses of the local flora in Chenab riverine area, Punjab province Pakistan. J. Ethnobiol. Ethnomed..

[27] Khalid M., Hassani D., Bilal M., Butt Z.A., Hamayun M., Ahmad A., Huang D., Hussain A. (2017). Identification of oral cavity biofilm forming bacteria and determination of their growth inhibition by Acacia arabica, *Tamarix aphylla* L. and Melia azedarach L. medicinal plants. Arch. Oral Biol..

[28] Uzair M., Ijaz A.S., Khan T.R. (2014). Survey of Ethno-Medicinal Weeds of District Rajhan Pur. Indian Res. J. Pharm. Sci..

[29] Suleiman M.H.A. (2019). Ethnobotanical, Phytochemical, and Biological Study of Tamarix aphylla and Aerva javanica Medicinal Plants Growing in the Asir Region, Saudi Arabia. Trop. Conserv. Sci..

[30] Kamal M., Wazir S.M., Hassan M., Saad M.S., Khan U., Muhammad A., Taj S. (2009). Ethnobotanically Important Plants of District Bannu, Pakistan. J. Plant Sci..

[31] Eddouks M., Maghrani M., Lemhadri A., Ouahidi M.-L., Jouad H. (2002). Ethnopharmacological survey of medicinal plants used for the treatment of diabetes mellitus, hypertension and cardiac diseases in the south-east region of Morocco (Tafilalet). J. Ethnopharmacol..

[32] Merzouki A., Ed-derfoufi F., Molero Mesa J. (2000). Contribution to the knowledge of Rifian traditional medicine. II: Folk medicine in Ksar Lakbir district (NW Morocco). Fitoterapia.

[33] Pittler M. (2010). A Guide to Medicinal Plants. Focus Altern. Complementary Ther..

[34] Tahraoui A., El-Hilaly J., Israili Z.H., Lyoussi B. (2007). Ethnopharmacological survey of plants used in the traditional treatment of hypertension and diabetes in south-eastern Morocco (Errachidia province). J. Ethnopharmacol..

[35] Al-Fatimi M. (2019). Ethnobotanical survey of medicinal plants in central Abyan governorate, Yemen. J. Ethnopharmacol..

[36] Shah A., Rahim S., Bhatti K.H., Khan A., Din N., Imran M., Mohsin M., Ishtiaq M., Nabila A., Ansari A. (2015). Ethnobotanical study and conservation status of trees in the district Sargodha, Punjab, Pakistan. Phyton.

[37] Bahadur S., Khan M.S., Shah M., Shuaib M., Ahmad M., Zafar M., Begum N., Gul S., Ashfaq S., Mujahid I. (2020). Traditional usage of medicinal plants among the local communities of Peshawar valley, Pakistan. Acta Ecol. Sin..

[38] Saeed Khattak N., Nouroz F., Ur Rahman I., Noreen S. (2015). Ethno veterinary uses of medicinal plants of district Karak, Pakistan. J. Ethnopharmacol..

[39] Yaseen G., Ahmad M., Zafar M., Sultana S., Kayani S., Cetto A.A., Shaheen S. (2015). Traditional management of diabetes in Pakistan: Ethnobotanical investigation from Traditional Health Practitioners. J. Ethnopharmacol..

[40] Hammiche V., Maiza K. (2006). Traditional medicine in Central Sahara: Pharmacopoeia of Tassili N’ajjer. J. Ethnopharmacol..

[41] Alzweiri M., Al Sarhan A., Mansi K., Hudaib M., Aburjai T. (2011). Ethnopharmacological survey of medicinal herbs in Jordan, the Northern Badia region. J. Ethnopharmacol..

[42] Brock J.H. (1994). *Tamarix* spp. (Salt Cedar), an invasive exotic woody plant in arid and semi-arid riparian habitats of western USA. Ecol. Manag. Invasive Riverside Plants.

[43] Horton J.S. Notes on the Introduction of Deciduous Tamarisk. https://gifiloqokawom.pdfleadership.icu/notes-on-the-introduction-of-deciduous-tamarisk-book-21438vb.php.

[44] Jasiem T.M., Nasser N.M., Al-Bazaz H.K. (2019). *Tamarix aphylla* L.: A review. Res. J. Pharm. Technol..

[45] Nawwar M.A.M., Hussein S.A.M. (1994). Gall polyphenolics of Tamarix aphylla. Phytochemistry.

[46] Nag G., De B. (2011). Acetylcholinesterase inhibitory activity of Terminalia chebula, Terminalia bellerica and Emblica officinalis and some phenolic compounds. Int. J. Pharm. Pharm. Sci..

[47] Sharma S.K., Parmar V.S., Shanna S.K., Parmar V.S. (1998). Novel constituents of Tamarix species. J. Sci. Ind. Res..

[48] Souliman A.M.A., Barakat H.H., El-Mousallamy A.M.D., Marzouk M.S.A., Nawwar M.A.M. (1991). Phenolics from the bark ofTamarix aphylla. Phytochemistry.

[49] Nawwar M.A.M., Hussein S.A.M., Buddrus J., Linscheid M. (1994). Tamarixellagic acid, an ellagitannin from the galls of Tamarix aphylla. Phytochemistry.

[50] Shafaghat A. (2010). Phytochemical investigation of quranic fruits and plants. J. Med. Plants.

[51] Orabi M.A.A., Taniguchi S., Sakagami H., Yoshimura M., Yoshida T., Hatano T. (2013). Hydrolyzable tannins of tamaricaceous plants. V. Structures of monomeric-trimeric tannins and cytotoxicity of macrocyclic-type tannins isolated from tamarix nilotica (1). J. Nat. Prod..

[52] Ksouri R., Megdiche W., Falleh H., Trabelsi N., Boulaaba M., Smaoui A., Abdelly C. (2008). Influence of biological, environmental and technical factors on phenolic content and antioxidant activities of Tunisian halophytes. Comptes Rendus—Biol..

[53] Ishak M.S., El-Sissi H.I., El-Sherbieny A.E., Nawwar M.A. (1972). Tannins and polyphenolics of the galls of Tamarix aphylla. II. Planta Med..

[54] Mahfoudhi A., Grosso C., Gonçalves R.F., Khelifi E., Hammami S., Achour S., Trabelsi-Ayadi M., Valentão P., Andrade P.B., Mighri Z. (2016). Evaluation of Antioxidant, Anticholinesterase, and Antidiabetic Potential of Dry Leaves and Stems in Tamarix aphylla Growing Wild in Tunisia. Chem. Biodivers..

[55] Baaka N., Mahfoudhi A., Haddar W., Mhenni M.F., Mighri Z. (2017). Green dyeing process of modified cotton fibres using natural dyes extracted from *Tamarix aphylla* (L.) Karst. leaves. Nat. Prod. Res..

[56] Orfali R.S., Ebada S.S., El-Shafae A.M., Al-Taweel A.M., Lin W.H., Wray V., Proksch P. (2009). 3-O-trans-caffeoylisomyricadiol: A new triterpenoid from Tamarix nilotica growing in Saudi Arabia. Z. Nat.—Sect. C J. Biosci..

[57] Nawwar M.A.M., El Sherbeiny A.E.A., El Ansari M.A. (1975). Plant constitutents ofTamarix aphylla flowers (Tamaricaceae). Experientia.

[58] Chen L., Deng H., Cui H., Fang J., Zuo Z., Deng J., Li Y., Wang X., Zhao L. (2018). Inflammatory responses and inflammation-associated diseases in organs. Oncotarget.

[59] Wahab S., Ahmad M.F., Hussain A., Usmani S., Shoaib A., Ahmad W. (2021). Effectiveness of Azithromycin as add-on Therapy in COVID-19 Management. Mini-Rev. Med. Chem..

[60] Karin M., Clevers H. (2016). Reparative inflammation takes charge of tissue regeneration. Nature.

[61] Leonardi G.C., Accardi G., Monastero R., Nicoletti F., Libra M. (2018). Ageing: From inflammation to cancer. Immun. Ageing.

[62] Wongrakpanich S., Wongrakpanich A., Melhado K., Rangaswami J. (2018). A Comprehensive Review of Non-Steroidal Anti-Inflammatory Drug Use in The Elderly. Aging Dis..

[63] Gadallah A.S., Mujeeb-Ur-Rehman, Atta-Ur-Rahman, Yousuf S., Atia-Tul-Waha, Jabeen A., Swilam M.M., Khalifa S.A.M., El-Seedi H.R., Iqbal Choudhary M. (2020). Anti-inflammatory principles from *Tamarix aphylla* L.: A bioassay-guided fractionation study. Molecules.

[64] Alsayari A., Wahab S. (2021). Genus Ziziphus for the treatment of chronic inflammatory diseases. Saudi J. Biol. Sci..

[65] Guo S., DiPietro L.A. (2010). Critical review in oral biology & medicine: Factors affecting wound healing. J. Dent. Res..

[66] Gallelli G., Cione E., Serra R., Leo A., Citraro R., Matricardi P., Di Meo C., Bisceglia F., Caroleo M.C., Basile S. (2020). Nano-hydrogel embedded with quercetin and oleic acid as a new formulation in the treatment of diabetic foot ulcer: A pilot study. Int. Wound J..

[67] Pereira R.F., Bártolo P.J. (2016). Traditional Therapies for Skin Wound Healing. Adv. Wound Care.

[68] Abbas B., Al-Qarawi A.A., Al-Hawas A. (2002). The ethnoveterinary knowledge and practice of traditional healers in Qassim region, Saudi Arabia. J. Arid. Environ..

[69] Sajid Ali M., Sarfaraz Alam M., Ahmad S., Ali M., Ahsan W., Siddiqui M.R., Salahuddin Ansari M., Shamim M., Ali M.D. (2019). Wound healing activity of alcoholic extract of *Tamarixaphylla* L. On animal models. Biomed. Pharmacol. J..

[70] Soliman Yu H., Ibrahim Al S. (2011). Anti-inflammatory and Wound Healing Activities of Herbal Gel Containing an Antioxidant Tamarix aphylla Leaf Extract. Int. J. Pharmacol..

[71] Hussain A., Rizvi A., Wahab S., Ansari S., Hussain S., Zareen I. (2011). Antibacterial Screening of the Bark of *Adenanthera pavonina* (L.). Int. J. Biomed. Res..

[72] Hussain A., Wahab S., Zarin I., Hussain M.D.S. (2010). Antibacterial Activity of the Leaves of Coccinia indica (W. and A) Wof India. Biol. Res..

[73] Ahmad M.D.F., Wahab S., Ali Ahmad F., Intakhab Alam M., Ather H., Siddiqua A., Amir Ashraf S., Abu Shaphe M., Idreesh Khan M., Ali Beg R. (2021). A novel perspective approach to explore pros and cons of face mask in prevention the spread of SARS-CoV-2 and other pathogens. Saudi Pharm. J..

[74] El-Zayat M.M., Eraqi M.M., Alfaiz F.A., Elshaer M.M. (2021). Antibacterial and antioxidant potential of some Egyptian medicinal plants used in traditional medicine. J. King Saud Univ.-Sci..

[75] Verma R., Pavithra P., Janani V., Charumathi K., Indumathy R., Potala S. (2010). Antibacterial activity of plants used in Indian herbal medicine. Int. J. Green Pharm..

[76] Dorman H.J.D., Deans S.G. (2000). Antimicrobial agents from plants: Antibacterial activity of plant volatile oils. J. Appl. Microbiol..

[77] Adnan M., Tariq A., Bibi R., AbdElsalam N., Rehman H., Murad W., Ahmad S., Israr M., Sabahat S., Ullah R. (2015). Antimicrobial potential of alkaloids and flavonoids extracted from *Tamarix aphylla* leaves against common human pathogenic bacteria. Afr. J. Tradit. Complement. Altern. Med..

[78] Taghipour M.T., Nameni R., Taghipour M., Ghorat F. (2020). Phytochemical Analysis and Antimicrobial Activity of Ziziphus spina-christi and Tamarix aphylla Leaves’ Extracts as Effective Treatment for Coronavirus Disease 2019 (COVID-19). Thrita.

[79] Meot-Duros L., Le Floch G., Magné C. (2008). Radical scavenging, antioxidant and antimicrobial activities of halophytic species. J. Ethnopharmacol..

[80] Al Sobeai S.M. (2018). Anticancer, Cytotoxic Effect of Tamarix Aphylla, and Antibacterial Screening Efficiency against Multidrug-Resistant Human Pathogens. Asian J. Pharm. Clin. Res..

[81] Iqbal H., Ishfaq M., Abbas M.N., Ahmad I., Rehman A., Amin S.B., Shagufta B.I., Ullah M. (2015). In vitro Antimicrobial Study of Tamarix aphylla in View of Phytochemical Constituents. Pharmacologia.

[82] Saini R., Saini S., Sharma S. (2011). Biofilm: A dental microbial infection. J. Nat. Sci. Biol. Med..

[83] Diao M., Liang Y., Zhao J., Zhao C., Zhang J., Zhang T. (2021). Enhanced cytotoxicity and antioxidant capacity of kaempferol complexed with α-lactalbumin. Food Chem. Toxicol..

[84] del Valle P., García-Armesto M.R., de Arriaga D., González-Donquiles C., Rodríquez-Fernández P., Rúa J. (2016). Antimicrobial activity of kaempferol and resveratrol in binary combinations with parabens or propyl gallate against Enterococcus faecalis. Food Control.

[85] Sanglard D. (2002). Clinical relevance of mechanisms of antifungal drug resistance in yeasts. Enferm. Infecc. Microbiol. Clínica.

[86] Hay R.J., Johns N.E., Williams H.C., Bolliger I.W., Dellavalle R.P., Margolis D.J., Marks R., Naldi L., Weinstock M.A., Wulf S.K. (2014). The Global Burden of Skin Disease in 2010: An Analysis of the Prevalence and Impact of Skin Conditions. J. Investig. Dermatol..

[87] Ghazwani M., Hani U., Osmani R.A.M., Rahamathulla M., Begum M.Y., Wahab S., Gowda D.V., Ravikumar A.A., Kumar H.Y., Ather H. (2021). An Efficient Herbal Approach for Treating Fungal Infection in Cervical Cancer Patients by Developing and Optimizing a Vaginal Suppository. Int. J. Polym. Sci..

[88] Kumar Mishra K., Deep Kaur C., Kumar Sahu A., Panik R., Kashyap P., Prasad Mishra S., Dutta S. (2020). Medicinal Plants Having Antifungal Properties. Medicinal Plants—Use in Prevention and Treatment of Diseases [Working Title].

[89] Arif T., Bhosale J.D., Kumar N., Mandal T.K., Bendre R.S., Lavekar G.S., Dabur R. (2009). Natural products—Antifungal agents derived from plants. J. Asian Nat. Prod. Res..

[90] Bibi S., Afzal M., Khan M.B. (2015). Antifungal Activity of *Tamarix aphylla* (L.) Karst. Stem-bark Extract Against Some Pathogenic Fungi. Am.-Eurasian J. Agric. Environ. Sci..

[91] Caton J.G., Armitage G., Berglundh T., Chapple I.L., Jepsen S., Kornman K.S., Mealey B.L., Papapanou P.N., Sanz M., Tonetti M.S. (2018). A new classification scheme for periodontal and peri-implant diseases and conditions—Introduction and key changes from the 1999 classification. J. Clin. Periodontol..

[92] Mann J., Bernstein Y., Findler M. (2019). Periodontal disease and its prevention, by traditional and new avenues (Review). Exp. Ther. Med..

[93] Huang R., Li M., Gregory R.L. (2011). Bacterial interactions in dental biofilm. Virulence.

[94] Ahmad I., Wahab S., Nisar N., Dera A.A., Alshahrani M.Y., Abullias S.S., Irfan S., Alam M.M., Srivastava S. (2020). Evaluation of antibacterial properties of Matricaria aurea on clinical isolates of periodontitis patients with special reference to red complex bacteria. Saudi Pharm. J..

[95] Könönen E., Gursoy M., Gursoy U. (2019). Periodontitis: A Multifaceted Disease of Tooth-Supporting Tissues. J. Clin. Med..

[96] Marchesan J.T., Girnary M.S., Moss K., Monaghan E.T., Egnatz G.J., Jiao Y., Zhang S., Beck J., Swanson K.V. (2020). Role of inflammasomes in the pathogenesis of periodontal disease and therapeutics. Periodontology 2000.

[97] Orban B. (1955). Gingivitis. J. Periodontol..

[98] Trombelli L., Farina R., Silva C.O., Tatakis D.N. (2018). Plaque-induced gingivitis: Case definition and diagnostic considerations. J. Clin. Periodontol..

[99] Chen L.H. (2018). Nutritional Aspects of Aging.

[100] Anticholinesterase|Drug|Britannica. https://www.britannica.com/science/anticholinesterase.

[101] Merfort I., Buddrus J., Nawwar M.A.M., Lambert J. (1992). A triterpene from the bark of Tamarix aphylla. Phytochemistry.

[102] Szwajgier D., Borowiec K. (2012). Phenolic acids from malt are efficient acetylcholinesterase and butyrylcholinesterase inhibitors. J. Inst. Brew..

[103] Khan H., Pervaiz A., Intagliata S., Das N., Nagulapalli Venkata K.C., Atanasov A.G., Najda A., Nabavi S.M., Wang D., Pittalà V. (2020). The analgesic potential of glycosides derived from medicinal plants. DARU J. Pharm. Sci..

[104] Abo-Dola M., Lutfi M., Bakhiet A., Mohamed A. (2015). Anti-Inflammatory, Analgesic, Antipyretic and the Membrane-Stabilizing Effects of Tamarix aphylla Ethanolic Extract. Eur. J. Med. Plants.

[105] Balamurugan R., Duraipandiyan V., Ignacimuthu S. (2011). Antidiabetic activity of γ-sitosterol isolated from *Lippia nodiflora* L. in streptozotocin induced diabetic rats. Eur. J. Pharmacol..

[106] Nakrani M.N., Wineland R.H., Anjum F. (2020). Physiology, Glucose Metabolism.

[107] Mergenthaler P., Lindauer U., Dienel G.A., Meisel A. (2013). Sugar for the brain: The role of glucose in physiological and pathological brain function. Trends Neurosci..

[108] Bhagavan N.V., Ha C.-E. (2015). Carbohydrate Metabolism II. Essent. Med. Biochem..

[109] Association A.D. (2009). Diagnosis and classification of diabetes mellitus. Diabetes Care.

[110] Farrah T.E., Dhillon B., Keane P.A., Webb D.J., Dhaun N. (2020). The eye, the kidney, and cardiovascular disease: Old concepts, better tools, and new horizons. Kidney Int..

[111] Eizirik D.L., Colli M.L., Ortis F. (2009). The role of inflammation in insulitis and Β-cell loss in type 1 diabetes. Nat. Rev. Endocrinol..

[112] Wright E., Scism-Bacon J.L., Glass L.C. (2006). Oxidative stress in type 2 diabetes: The role of fasting and postprandial glycaemia. Int. J. Clin. Pract..

[113] Kooti W., Farokhipour M., Asadzadeh Z., Ashtary-Larky D., Asadi-Samani M. (2016). The role of medicinal plants in the treatment of diabetes: A systematic review. Electron. Physician.

[114] Balamurugan R., Ignacimuthu S. (2011). Antidiabetic and hypolipidemic effect of methanol extract of *Lippia nodiflora* L. in streptozotocin induced diabetic rats. Asian Pac. J. Trop. Biomed..

[115] Kumar S., Kumar V., Prakash O. (2011). Antidiabetic, hypolipidemic and histopathological analysis of *Dillenia indica* (L.) leaves extract on alloxan induced diabetic rats. Asian Pac. J. Trop. Med..

[116] Jolles J.W., de Visser L., van den Bos R. (2011). Male Wistar rats show individual differences in an animal model of conformity. Anim. Cogn..

[117] Daisy P., Eliza J., Mohamed Farook K.A.M. (2009). A novel dihydroxy gymnemic triacetate isolated from Gymnema sylvestre possessing normoglycemic and hypolipidemic activity on STZ-induced diabetic rats. J. Ethnopharmacol..

[118] Garud M.S., Kulkarni Y.A. (2018). Gallic acid attenuates type I diabetic nephropathy in rats. Chem.-Biol. Interact..

[119] Obafemi T.O., Jaiyesimi K.F., Olomola A.A., Olasehinde O.R., Olaoye O.A., Adewumi F.D., Afolabi B.A., Adewale O.B., Akintayo C.O., Ojo O.A. (2021). Combined effect of metformin and gallic acid on inflammation, antioxidant status, endoplasmic reticulum (ER) stress and glucose metabolism in fructose-fed streptozotocin-induced diabetic rats. Toxicol. Rep..

[120] Wong T.S., Ismail H.F., Hashim Z., Majid F.A.A. (2019). Synergistic antihyperglycaemic effect of combination therapy with gallic acid and andrographolide in streptozotocin-induced diabetic rats. Biocatal. Agric. Biotechnol..

[121] Arfin S., Siddiqui G.A., Naeem A., Moin S. (2018). Inhibition of advanced glycation end products by isoferulic acid and its free radical scavenging capacity: An in vitro and molecular docking study. Int. J. Biol. Macromol..

[122] Ranilla L.G., Kwon Y.-I., Apostolidis E., Shetty K. (2010). Phenolic compounds, antioxidant activity and in vitro inhibitory potential against key enzymes relevant for hyperglycemia and hypertension of commonly used medicinal plants, herbs and spices in Latin America. Bioresour. Technol..

[123] Verma N., Behera B.C., Sharma B.O. (2012). Glucosidase Inhibitory and Radical Scavenging Properties of Lichen Metabolites Salazinic Acid, Sekikaic Acid and Usnic Acid Liken Metabolitleri Salazinik, Sekikaik ve Usnik Asitin Glikosidaz Engelleyici ve Radikal Süpürücü Özelliği Research Article. J. Biol. Chem. J. Biol. Chem..

[124] Ramkumar K.M., Thayumanavan B., Palvannan T., Rajaguru P. (2010). Inhibitory effect of Gymnema Montanum leaves on α-glucosidase activity and α-amylase activity and their relationship with polyphenolic content. Med. Chem. Res..

[125] Li D.Q., Qian Z.M., Li S.P. (2010). Inhibition of three selected beverage extracts on α-glucosidase and rapid identification of their active compounds using HPLC-DAD-MS/MS and biochemical detection. J. Agric. Food Chem..

[126] Önal S., Timur S., Okutucu B., Zihnioǧlu F. (2005). Inhibition of α-glucosidase by aqueous extracts of some potent antidiabetic medicinal herbs. Prep. Biochem. Biotechnol..

[127] You Q., Chen F., Wang X., Jiang Y., Lin S. (2012). Anti-diabetic activities of phenolic compounds in muscadine against alpha-glucosidase and pancreatic lipase. LWT—Food Sci. Technol..

[128] Yao X., Zhu L., Chen Y., Tian J., Wang Y. (2013). In vivo and in vitro antioxidant activity and α-glucosidase, α-amylase inhibitory effects of flavonoids from Cichorium glandulosum seeds. Food Chem..

[129] Atanasov A.G., Zotchev S.B., Dirsch V.M., Orhan I.E., Banach M., Rollinger J.M., Barreca D., Weckwerth W., Bauer R., Bayer E.A. (2021). Natural products in drug discovery: Advances and opportunities. Nat. Rev. Drug Discov..

[130] Rajagopalan P., Wahab S., Dera A., Chandramoorthy H., Irfan S., Patel A., Abullias S., Zaman G., Ahmad I. (2020). Anti-cancer activity of ethanolic leaf extract of Salvia officinalis against oral squamous carcinoma cells in vitro via caspase mediated mitochondrial apoptosis. Pharmacogn. Mag..

[131] Abaza M.S.I., Afzal M., Al-Attiyah R.J., Guleri R. (2016). Methylferulate from Tamarix aucheriana inhibits growth and enhances chemosensitivity of human colorectal cancer cells: Possible mechanism of action. BMC Complement. Altern. Med..

[132] Ferlay J., Soerjomataram I., Dikshit R., Eser S., Mathers C., Rebelo M., Parkin D.M., Forman D., Bray F. (2015). Cancer incidence and mortality worldwide: Sources, methods and major patterns in GLOBOCAN 2012. Int. J. Cancer.

[133] Wahab S., Alshahrani M.Y., Ahmad M.F., Abbas H. (2021). Current trends and future perspectives of nanomedicine for the management of colon cancer. Eur. J. Pharmacol..

[134] Wahab S., Annadurai S., Abullais S.S., Das G., Ahmad W., Ahmad M.F., Kandasamy G., Vasudevan R., Ali M.S., Amir M. (2021). Glycyrrhiza glabra (Licorice): A Comprehensive Review on Its Phytochemistry, Biological Activities, Clinical Evidence and Toxicology. Plants.

[135] Amin A.R., Kucuk O., Khuri F.R., Shin D.M. (2009). Perspectives for cancer prevention with natural compounds. J. Clin. Oncol..

[136] Siegel R., Naishadham D., Jemal A. (2012). Cancer statistics for Hispanics/Latinos, 2012. CA Cancer J. Clin..

[137] Newman D.J., Cragg G.M. (2012). Natural products as sources of new drugs over the 30 years from 1981 to 2010. J. Nat. Prod..

[138] Namasivayam N. (2011). Chemoprevention in experimental animals. Ann. N. Y. Acad. Sci..

[139] Romeilah R.M., El-Beltagi H.S., Shalaby E.A., Younes K.M., EL Moll H., Rajendrasozhan S., Mohamed H. (2021). Antioxidant and cytotoxic activities of *Artemisia monosperma* L. and *Tamarix aphylla* L. essential oils. Not. Bot. Horti Agrobot. Cluj-Napoca.

[140] Alhourani N., Kasabri V., Bustanji Y., Abbassi R., Hudaib M. (2018). Potential Antiproliferative Activity and Evaluation of Essential Oil Composition of the Aerial Parts of *Tamarix aphylla* (L.) H. Karst: A Wild Grown Medicinal Plant in Jordan. Evid.-Based Complement. Altern. Med..

[141] Muhammad H., Wazir S.M., Khan R.A. (2017). In Vitro Antioxidant, Antifungal and Cytotoxic Activities of Selected Medicinal Plants of District Bannu and Lakki Marwat. Stud. Ethno-Med..

[142] Khalid M., Bilal M., Munir H., Shah S.Z.H., Khurshid M., El-Shazly M., Iqbal H.M.N. (2020). In-vitro Evaluation of Anti-Bacterial, Anti-biofilm and Cytotoxic Activity of Naturally Inspired Juglans regia, Tamarix aphylla L., and Acacia modesta with Medicinal Potentialities. J. Pure Appl. Microbiol..

[143] El Ansari M.A., Nawwar M.A.M., El Dein A., El Sherbeiny A., El Sissi H.I. (1976). A sulphated kaempferol 7,4′-dimethyl ether and a quercetin isoferulylglucuronide from the flowers of Tamarix aphylla. Phytochemistry.

[144] Yusufoglu H.S., Alam A., Al-Howeemel A. (2015). Pharmacognostie and preliminary phytochemical standardization of Tainarix aphylla and Ziziphus nummularia growing in Saudi Arabia. Asian J. Biol. Life Sci..

[145] Orabi M.A.A., Yoshimura M., Amakura Y., Hatano T. (2015). Ellagitannins, gallotannins, and gallo-ellagitannins from the galls of Tamarix aphylla. Fitoterapia.

[146] Nawwar M., Swilam N., Hashim A., Al-Abd A., Abdel-Naim A., Lindequist U. (2013). Cytotoxic isoferulic acidamide from Myricaria germanica (Tamaricaceae). Plant Signal. Behav..

[147] Nicolini F., Burmistrova O., Marrero M.T., Torres F., Hernández C., Quintana J., Estévez F. (2014). Induction of G 2 /M phase arrest and apoptosis by the flavonoid tamarixetin on human leukemia cells. Mol. Carcinog..

[148] Orabi K.Y., Abaza M.S., El Sayed K.A., Elnagar A.Y., Al-Attiyah R., Guleri R.P. (2013). Selective growth inhibition of human malignant melanoma cells by syringic acid-derived proteasome inhibitors. Cancer Cell Int..

[149] AMA A. (2017). Tamarix nilotica (Ehrenb) Bunge: A Review of Phytochemistry and Pharmacology. J. Microb. Biochem. Technol..

[150] Martins J., Brijesh S. (2018). Phytochemistry and pharmacology of anti-depressant medicinal plants: A review. Biomed. Pharmacother..

[151] Nasri H., Hedayatollah S. (2013). Toxicity and safety of medicinal plants. J. HerbMed. Pharmacol..

[152] Wahab S., Ahmad I., Irfan S., Baig M.H., Farouk A.-E., Dong J.-J. (2021). Use of Natural Compounds as a Potential Therapeutic Agent Against COVID-19. Curr. Pharm. Des..

[153] Alshahrani M.Y., Rafi Z., Alabdallah N.M., Shoaib A., Ahmad I., Asiri M., Zaman G.S., Wahab S., Saeed M., Khan S. (2021). A comparative antibacterial, antioxidant, and antineoplastic potential of *rauwolfia serpentina* (L.) leaf extract with its biologically synthesized gold nanoparticles (r-aunps). Plants.

[154] Ashour O.M., Abdel-Naim A.B., Abdallah H.M., Nagy A.A., Mohamadin A.M., Abdel-Sattar E.A. (2012). Evaluation of the Potential Cardioprotective Activity of Some Saudi Plants against Doxorubicin Toxicity. Z. Nat. C.

